# Anti-Quorum-Sensing Activity of Tryptophan-Containing Cyclic Dipeptides

**DOI:** 10.3390/md20020085

**Published:** 2022-01-19

**Authors:** Yinglu Wang, Qian Zheng, Li Li, Lile Pan, Hu Zhu

**Affiliations:** Engineering Research Center of Industrial Biocatalysis, Fujian Provincial Key Laboratory of Advanced Materials Oriented Chemical Engineering, Fujian Provincial Key Laboratory of Polymer Materials, College of Chemistry and Materials Science, Fujian Normal University, Fuzhou 350007, China; wangyl@fjnu.edu.cn (Y.W.); qsxzhengq@163.com (Q.Z.); lilifnju@163.com (L.L.); panlljxgz@163.com (L.P.)

**Keywords:** anti-quorum sensing, cyclic dipeptides, tryptophane, *Pseudomonas aeruginosa*, biofilm, bacterial adhesion

## Abstract

Quorum sensing (QS) can regulate the pathogenicity of bacteria and the production of some virulence factors. It is a promising target for screening to find anti-virulence agents in the coming post-antibiotics era. Cyclo (*L*-Trp-*L*-Ser), one variety of cyclic dipeptides (CDPs), isolated from a marine bacterium *Rheinheimera aquimaris*, exhibited anti-QS activity against *Chromobacterium violaceum* CV026 and *Pseudomonas aeruginosa* PAO1. Unlike the CDPs composed of phenylalanine or tyrosine, the anti-QS activity has been widely studied; however, cyclo (*L*-Trp-*L*-Ser) and derivatives, containing one tryptophan unit and one non-aromatic amino acid, have not been systematically explored. Herein, the cyclo (*L*-Trp-*L*-Ser) and seven derivatives were synthesized and evaluated. All tryptophane-contained CDPs were able to decrease the production of violacein in *C.* *violaceum* CV026 and predicted as binding within the same pocket of receptor protein CviR, but in lower binding energy compared with the natural ligand C_6_HSL. As for *P. aeruginosa* PAO1, owning more complicated QS systems, these CDPs also exhibited inhibitory effects on pyocyanin production, swimming motility, biofilm formation, and adhesion. These investigations suggested a promising way to keep the tryptophan untouched and make modifications on the non-aromatic unit to increase the anti-QS activity and decrease the cytotoxicity, thus developing a novel CDP-based anti-virulence agent.

## 1. Introduction

Quorum sensing (QS) is a well-known cell-to-cell communication in microorganisms [1], through which bacteria regulate cell density-dependent behavior, such as secreting virulence factors, motility, and biofilm formation, aids bacteria in infections and drug resistance [2]. In recent years, to combat the growing seriousness of antibiotic resistance, more focus has been turned to disarming bacterial pathogenicity by decreasing their virulence factors [3,4] and biofilm formation [5,6]. Inhibition of the QS system, which can disrupt bacterial communication, thus affecting the secretion of virulence factors and biofilm formation, rather than kill the bacteria, will pose less survival pressure on bacteria and inhibit their evolution into drug-resistant strains and is attracting much attention [7,8]. In the QS system, small molecules, named autoinducers (AIs), act as signals, through which bacteria can detect the surrounding population [9]. When the concentration of AI reaches a certain threshold, it binds with the receptor and then activates the expression of the corresponding genes. QS inhibitors (QSIs) can block the communication through three general strategies: (i) interfering signal molecules synthase to decrease the concentration of autoinducers; (ii) inducing the degradation of autoinducers; (iii) inhibiting the binding between AIs and receptor proteins [10].

**Figure 1:**
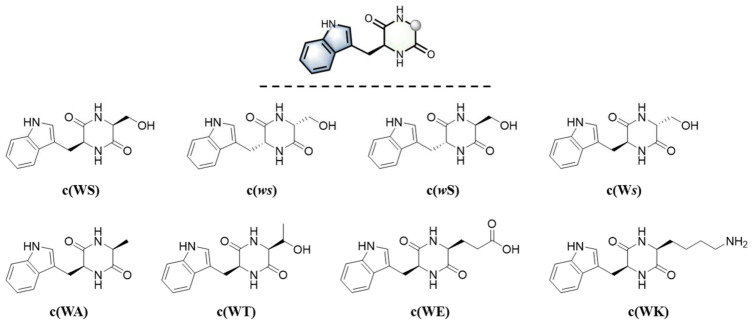
Cyclic dipeptides for the test of anti-QS activity in this work.

Our previous studies focus on seeking anti-QS agents from marine microorganisms to fight against gram-negative bacteria *Pseudomonas* *aeruginosa*. *P*. *aeruginosa* is a well-known opportunistic pathogen in hospitals and poses a huge threat to immunodeficiency patients [11,12,13]. Numerous studies have shown that the expression of virulence factors for pathogenicity in *P*. *aeruginosa* is regulated by its QS system [14,15]. In the past few years, several known compounds, including some cyclic dipeptides, proved to exhibit anti-QS activity, such as chrysin, tyrosol, cyclo (*L*-Tyr-*L*-Pro) from *Penicillium chrysogenum* [16,17,18], cyclo (*L*-Trp-*L*-Ser) from *Rheinheimera aquimaris* [19], and cyclo (*L*-Phe-*L*-Pro) from *Sphingomonas* sp. WG [20]. The stable and structurally diverse framework of cyclic dipeptides with anti-QS activity attracted our interests. To date, the mechanism of how CDPs express inhibition over QS remains undefined [21,22], but it was revealed that most reported anti-QS and biofilm inhibiting CDPs contained phenylalanine, tyrosine, and proline [23]. Grenier and co-workers synthesized a variety of cyclic dipeptides containing the three amino acids above using the solid-phase synthesis strategy. They evaluated their antibiofilm and anti-adherence ability against oral pathogenic bacteria *S. mutans* and fungus *C. albicans* [24] and found that only the CDPs, both arms of which were aromatic residues, displayed potent activities against both *S. mutans* and *C. albicans*. By contrast, tryptophane-containing CDPs’ antimicrobial or anti-QS ability was less studied. We isolated cyclo (L-Trp-L-Ser) from *R. aquimaris*, which was identified as an anti-QS agent using a bioassay-guided method [19]: It not only decreased the QS-mediated virulence factors production of phenotype *Chromobacterium violaceum* CV026 and *P. aeruginosa* PAO1 in a dose-dependent manner but also exhibited a good activity of antibiofilm formation against *P. aeruginosa* PAO1. Compared with cyclo (*L*-Tyr-*L*-Pro) [18] and cyclo (*L*-Phe-*L*-Pro) [20], this CDP showed stronger inhibition of biofilm formation of *P. aeruginosa* PAO1. Furthermore, it would be easier to make structural modifications to expand the anti-QS relative chemical space. Therefore, to evaluate the effect of stereochemistry and side-chain differences on the anti-QS activity, especially for their anti-biofilm and anti-adhesion ability against *P. aeruginosa* PAO1, we employed a three-step synthetic sequence to get prepared cyclo (*L*-Trp-*L*-Ser) and seven derivatives (Figure 1).

## 2. Results

### 2.1. Antimicrobial Activity

**Figure 2:**
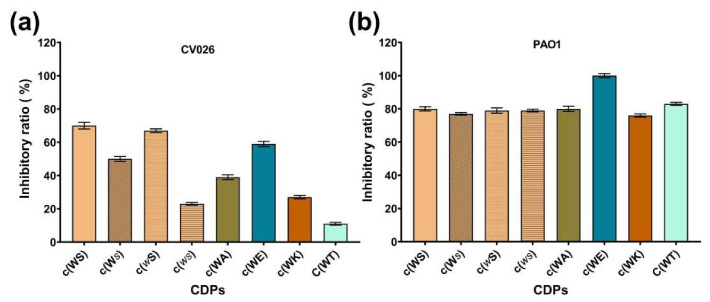
The inhibitory ratio of eight synthetic CDPs at the concentration of 15 mM against (**a**) *C. violaceum* CV026, and (**b**) *P. aeruginosa* PAO1.

**Figure 3:**
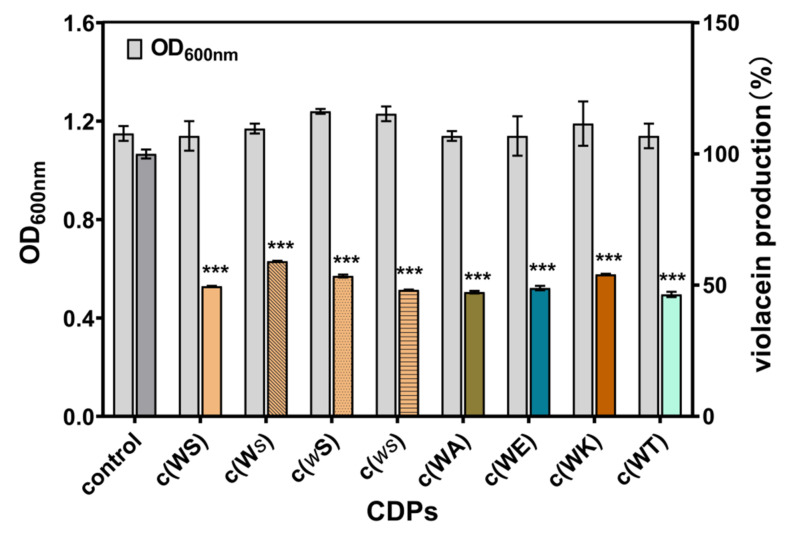
Effects of eight synthetic CDPs at the concentration of 1 mM on violacein production of *C. violaceum* CV026. Differences in mean absorbance were compared to the untreated control and considered significant when *** *p* < 0.001, according to the ANOVA.

**Figure 4:**
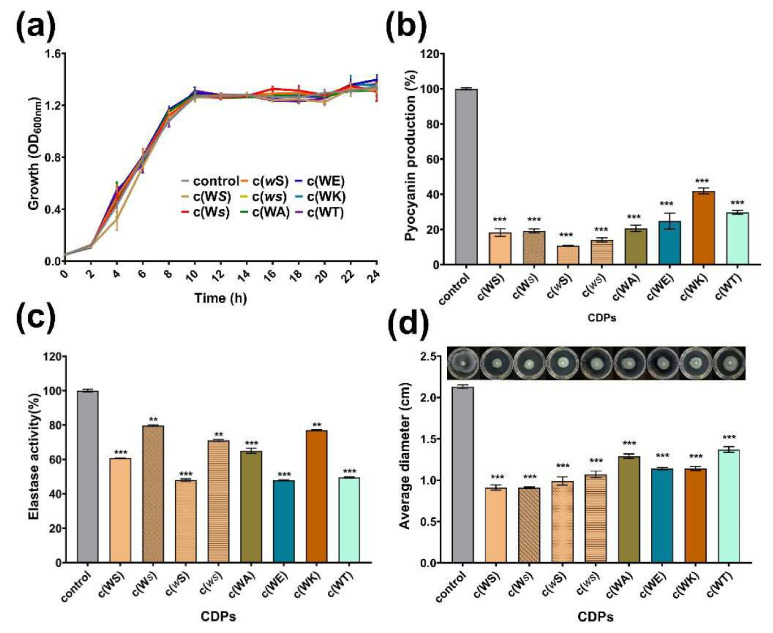
Effects of eight synthetic CDPs at the concentration of 1 mM on the production of virulence factors of *P. aeruginosa* PAO1. (**a**) The bacterial growth curve; (**b**) the effect of CDPs on pyocyanin production; (**c**) the effect of CDPs on elastase activity of PAO1; (**d**) the effect of CDPs on the swimming of PAO1. DMSO (1%, *v*/*v*) was served as the control. Each experiment was performed at least in triplicate. Differences in mean absorbance were compared to the untreated control and considered significant when ** *p* < 0.01, *** *p* < 0.001, according to ANOVA.

The MICs of the synthesized CDPs against *C. violaceum* CV026 and *P. aeruginosa* PAO1 were determined in MHB and M63 medium. However, except c(WE), MICs of all tested CDPs against the two strains were higher than 15 mM, above which they could not be tested because of the poor solubility. The inhibitory efficiency of CDPs against *C. violaceum* CV026 and *P. aeruginosa* PAO1 in M63 medium was illustrated in Table 1 and Figure 2. The MIC of c(WE) against *P. aeruginosa* PAO1 was 6.3 mM in M63 medium, while in MHB, it didn’t inhibit the bacterial growth significantly. According to our previous study about the anti-QS evaluation of isolated c(WS) [19], we chose 1 mM, at a concentration at which the bacterial growths were barely affected (Figure 3 and Figure 4a), as the maximum test concentration in this work. To avoid the precipitation of these CDPs during the experiment, dimethyl sulfoxide (DMSO) was used as a co-solvent at a final concentration of 1% (*v*/*v*), which we had previously confirmed does not impact the bacterial growth.

**Table 1:** The inhibitory efficiency of eight synthetic CDPs (15 mM) against *C. violaceum* CV026 and *P. aeruginosa* PAO1 in M63 medium. Different letters indicate statistical significance between groups by Tukey’s HSD test at *p* < 0.05.

Compound	Inhibitory Ratio (%)	
	CV026	PAO1
c(WS)	70% ^a^	80% ^a^
c(*ws*)	23% ^b^	79% ^a^
c(W*s*)	50% ^a^	77% ^a^
c(*w*S)	67% ^a^	79% ^a^
c(WA)	39% ^b^	80% ^a^
c(WT)	11% ^b^	83% ^a^
c(WK)	27% ^b^	76% ^a^
c(WE)	59% ^a^	100% ^a^

Note: This table uses the letters a and b to show statistically significant differences between inhibitory ratios in mean comparisons. If there are no significant differences between two inhibitory ratios, they get the same letter (a:a or b:b). If there are significant differences between two inhibitory ratios, they get the different letters (a:b).

### 2.2. Anti-QS Ability against C. violaceum CV026

*C. violaceum* CV026 is a typical model bacterium for anti-QS agent screening, which lacks autoinducer synthase gene *cviI*. Exogenous autoinducer C_6_HSL can induce the production of violacein. The anti-QS ability of CDPs could be confirmed by measuring violacein yield. As shown in Figure 3, at a final concentration of 1 mM, all CDPs inhibited violacein production by 40–60%, while the bacterial growth was not affected.

Molecular docking was performed to investigate the probable interaction between CDPs and QS-related receptor protein CviR in *C. violaceum*. The autoinducer C_6_HSL was docked in advance as a control and to establish a credible docking method. The results suggested that all CDPs can bind at the same pocket competitively with the C_6_HSL in CviR with lower binding energy. The detailed data about binding energy and hydrogen bonding interactions were illustrated in Table 2 (the images were shown in Figure S1). Four hydrogen bonds were found between the natural ligand C_6_HSL and CviR, which are highly consistent with the reported X-ray structure [25]. The c(WS) and its enantiomer c(*ws*) were found to form a hydrogen bond with Tyr88 and Ser 155 of CviR, while its isomers c(W*s*) formed a hydrogen bond with Tyr80 instead of Ser155.

Upon replacement of the hydroxyl group with H, the binding energy of c(WA) was raised slightly, while three hydrogen bonds were found. The more steric hindered c(WT) formed the same hydrogen bonds, as C_6_HSL with CviR, and the binding energy was lower. The charged CDPs, c(WK) and c(WE), bound with the protein with lower energy and also formed hydrogen bonds with Tyr88 and Ser155. Interestingly, no hydrogen bonds were found between c(*w*S) or the receptor protein.

**Table 2:** Detail docking information of CviR with autoinducer C_6_HSL and tryptophan-containing CDPs.

Molecules	Binding Energy (kcal/mol)	Hydrogen Bonding Interactions
C_6_HSL	−6.04	Asp97, Ser155, Trp84, Tyr80
c(WS)	−7.83	Tyr88, Ser155
c(*ws*)	−7.88	Tyr88, Ser155
c(W*s*)	−7.78	Tyr88, Tyr80
c(*w*S)	−7.52	No hydrogen bonds formed
c(WA)	−7.56	Tyr88, Trp84, Ser155
c(WT)	−7.90	Trp84, Ser155, Asp97, Tyr80
c(WK)	−8.14	Tyr88, Ser155
c(WE)	−8.09	Tyr88, Ser155

### 2.3. Inhibition on Production of Virulence Factors of P. aeruginosa PAO1

Pyocyanin is one of the QS-regulated virulence factors that *P. aeruginosa* secrete. All tested CDPs decreased the yield of pyocyanin dose-dependently at a concentration ranging from 0.1 to 1 mM (Figure S2). As shown in Figure 4a,b, at a concentration of 1 mM, which is the maximum treated concentration, CDPs suppressed the production of pyocyanin obviously, but did not affect the bacterial growth, especially for c(WE), c(WT), c(*w*S), c(W*s*), and c(*ws*), which remarkably reduced the yield of pyocyanin by 75%, 70%, 89%, 81%, and 86%, respectively. Elastase activity, another important virulence factor regulated by the QS system of *P. aeruginosa*, was assessed using elastin-Gongo red. The c(WS) can decrease the elastase activity by 39% at a concentration of 1 mM, which was similar to the result of the isolated activity [19]. Furthermore, c(WE), c(WT), and c(*w*S) seemed more efficient in suppressing the elastase activity (Figure 4b). The inhibition rate of elastase activity was proportional to CDP concentration (Figure S3).

Swimming motility, which is also regulated by the QS system in *P. aeruginosa*, plays an important role in expressing full virulence and colonization. As shown in Figure 4c, the swimming diameter of *P. aeruginosa* PAO1 was shrunk by 36–57% after being treated with CDPs at a concentration of 1 mM.

### 2.4. Inhibition on Biofilm and Adhesion of P. aeruginosa PAO1

**Figure 5:**
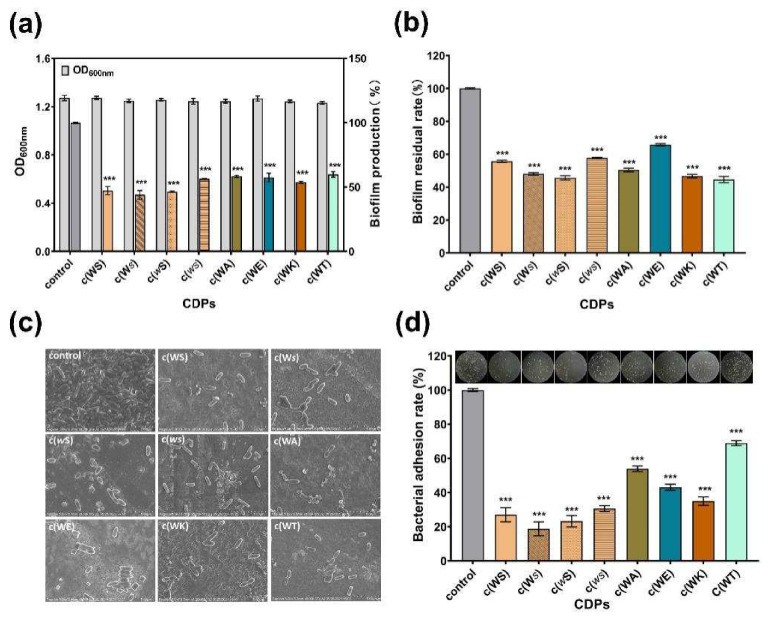
Effects of eight synthetic CDPs at a concentration of 1 mM on biofilm in *P. aeruginosa* PAO1. (**a**) The effects of CDPs on biofilm formation. (**b**) The effects of CDPs on the disruption of surface-established biofilms. (**c**) SEM images of the *P.aeruginosa* PAO1 biofilm, which was treated with CDPs. (**d**) The effects of CDPs on PAO1 adhesion. DMSO (1%, *v*/*v*) was served as the control. Differences in mean absorbances were compared to the untreated control and considered significant when *** *p* < 0.001, according to ANOVA.

The biofilm formation of *P. aeruginosa*, which was always considered to be QS-regulated, is complex and associated with many persistent infections and increased antibiotic resistance. All tested CDPs exhibited anti-biofilm activity against *P. aeruginosa* PAO1 in a dose-dependent manner (Figure S4). The c(WS) and its isomers, c(*w*S) and c(W*s*), inhibited the biofilm formation by 53%, 54%, and 56%, respectively, displaying more efficiency than other cyclic dipeptides, at a concentration of 1 mM (Figure 5a). Moreover, these CDPs decreased the mature biofilm by 40–56% (Figure 5b), showing a similar ability in anti-biofilm formation. Interestingly, c(WT), c(WA), and c(WK) exhibited higher biofilm elimination ability (56%, 50%, and 53%, respectively), while their anti-biofilm formation capability was not superior. Bacteria and biofilm morphologies observed by SEM (Figure 5c) indicated that the untreated bacterial cells were protected with the dense biofilm matrix, and treatment with CDPs decreased the biofilm formation and exposed the bacterial cells. Meanwhile, the bacterial cells were entirely observed as shot rods, indicating that bacterial cells were not destroyed under treatment with these CDPs.

Surface adhesion is a critical initial step that enables the establishment of biofilms. Therefore, the effects of CDPs on the adhesion of *P. aeruginosa* PAO1 to an abiotic surface were characterized. Figure 5d shows that the PAO1 cells, which adhered to the surface of the 96-well microplates, were sharply decreased in the groups treated with CDPs in comparison with the control group. Thus, this suggests that the CDPs obstructed the interactions between the abiotic surface and *P. aeruginosa* PAO1. Notably, isomers of cyclo (Trp-Ser) reduced the adhesion of *P. aeruginosa* PAO1 to the PVC surface by approximately three quarters.

### 2.5. Inhibition on QS-Regulated Genes of P. aeruginosa PAO1

**Figure 6:**
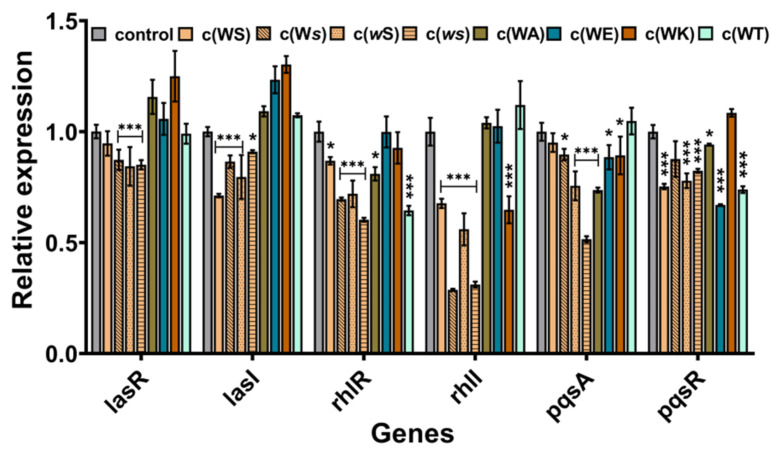
The effects of CDPs on genes expression *P. aeruginosa* PAO1. The *rpo*D was used as an internal standard. DMSO (1%, *v*/*v*) was served as the control. Differences in mean absorbance were compared to the untreated control and considered significant when * *p* < 0.05, *** *p* < 0.001, according to ANOVA.

To explore the probable anti-QS mechanism of these CDPs, the real-time RT-PCR was implemented to investigate the effects on QS genes expression. Although these CDPs are structurally alike and contain a diketopiperazine core moiety and indole group, they appeared to affect the expression of different QS genes. As shown in Figure 6, the isomers inhibited the expression of each gene to some extent, especially for that baring D-conformation Ser, which can suppress the expression of *rhl*I by 69% and 72%. However, the affection of the four analogs on the genes’ expression was varying. All of them seemed not to inhibit but promote the expression of genes in the *las* system. The sterically hindered c(WT) mainly inhibited the expression of *rhl*R and *pqs*R. The c(WA), which substitutes the hydroxyl group to hydrogen, mainly influenced the *pqs*A. The positively charged c(WK) decreased the expression of *rhl*I, while the negatively charged c(WE) mostly downregulated the expression of *pqs*R.

### 2.6. Hemolysis and Cytotoxicity

**Figure 7:**
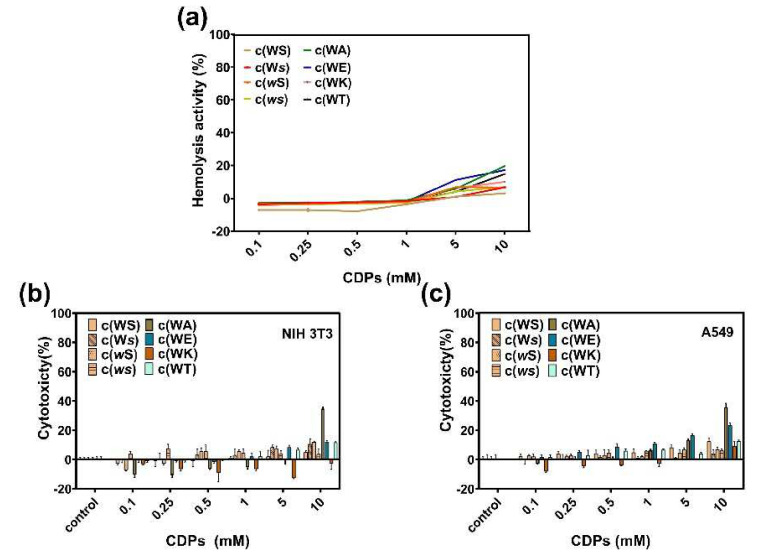
(**a**) Hemolysis of sheep red blood cells in the presence of CDPs over a broad range of concentrations (0.1−10 mM). Cytotoxicity of CDPs to NIH 3T3 cells (**b**) and A549 cells (**c**). Percent cytotoxicity relative to controls is represented as mean ± standard deviation of three biological replicates.

Hemolysis in the presence of these CDPs was evaluated by calculating the relative hemoglobin released at different CDPs concentrations compared with 0.1% Triton X-100, which can cause 100% hemolysis. All tested CDPs showed little hemolytic activity at a concentration of 1 mM (Figure 7a), and when the concentration was raised to 10 mM, c(WA), c(WT), and c(WE) caused more than 10% hemolysis. We estimate that the enhanced hydrophobicity of a CDP can increase its hemolysis.

To understand the toxicity of these CDPs to mammalian cells, cytotoxicity of CDPs on A549 and NIH-3T3 were investigated using the MTT method. Figure 7b,c indicates that, at concentrations lower than 1 mM, all CDPs displayed little toxicity to mammalian cells.

## 3. Discussion

To address bacterial infections, it is essential to discover or modify anti-QS compounds. The QS system of *P. aeruginosa* has been extensively studied and indicated as a promising target for developing antimicrobial drugs against this pathogen [15].

As one kind of stable secondary metabolites, numerous cyclic dipeptides were discovered from microorganisms and demonstrated as inhibitors, including antitumor, antiviral, and antibacterial [26,27]. It was reported that many cyclic dipeptides, acting as a new class of QS signal, were able to modulate the LuxR-Type receptor activity [28,29,30]. For instance, cyclo (*L*-Phe-*L*-Pro) produced by *Vibrio vulnificus* could induce the expression of *V. fischeri lux* genes and modulate ToxR dependent genes’ expression of *Vibrio spp* [30]. Additionally, cyclo (*L*-Phe-*L*-Pro), cyclo (*L*-Tyr-*L*-Pro), and cyclo (*L*-Tyr-*D*-Pro), isolated from *Lactobacillus reuteri* RC-14, were proved to affect the Gram-positive *staphylococcal* QS system [31].

To further explore the structural and activity of the cyclo(*L*-Trp-*L*-Ser), which was isolated from marine bacterium *R. aquimaris* and displayed anti-QS activity against typical QS-mediated phenotype *C. violaceum* CV026 and *P. aeruginosa* PAO1, we designed and synthesized seven CDPs, including three isomers and four analogs of cyclo(*L*-Trp-*L*-Ser), and then investigated their anti-QS ability. Results from this work demonstrated that tryptophan-containing cyclic dipeptides exhibited potent anti-QS ability against gram-negative bacteria *C. violaceum* CV026 and *P. aeruginosa* PAO1, plus their MICs were much higher or even unmeasurable. All tested CDPs reduced the violacein production of *C. violaceum* CV026 in a similar capacity by about 50%. In silico analysis suggested that these CDPs bind to the same pocket with lower binding energy than the natural ligand, which may lead to a conformational change of the active protein and may decrease the expression of the regulated virulence factor.

*P. aeruginosa* is a well-known Gram-negative opportunistic bacterial pathogen, which has posed serious threats to patients with cystic fibrosis or immunodeficiency, as well as intubated patients. Recently, in *P. aeruginosa* infection treatment, more and more studies have been focused on the anti-virulence factors and anti-biofilm formation [8,15,32,33]. In terms of inhibiting *P. aeruginosa* PAO1 at the given concentration of 1 mM, all tested CDPs expressed inhibition over the production of QS-regulated virulence factors, biofilm formation, and adhesion, moderately, without affecting the bacterial growth. In comparison, the yield of pyocyanin decreased more significantly than the activity changes of elastase. The reason might stem from the difference in the regulators of these phenotypes. It was reported that elastase production is mainly regulated by LasR [34], while the secretion of pyocyanin could be affected by many QS systems, and the mutations in the *las*, *rhl*, or *pqs* systems would result in the loss of pyocyanin production [35]. On the other hand, their capacities for inhibiting certain virulence phenotypes are slightly varying. We attributed this phenomenon to the more complex QS systems that *P. aeruginosa* has.

Furthermore, all these CDPs showed little hemolysis and cytotoxicity to sheep red blood cells and mammalian cells A549 and NIH-3T3, which indicated that the structural modifications, including the reversal of stereochemistry, replacement of the hydroxyl with other functional groups, such as H, carboxyl, or amine, and increasement of the steric hindrance barely impact the toxicity.

In summary, we synthesized a series of tryptophane-containing cyclic dipeptides and evaluated the anti-QS activity. It was revealed that this kind of CDP could inhibit violacein production in *C. violaceum* CV026 as a competitive inhibitor of CviR and suppress QS-mediated phenotypes in *P. aeruginosa* PAO1 to a certain extent. Since the differences in how these CDPs acted against phenotypes in *P. aeruginosa* PAO1 were confirmed as slight, the configuration changes of CDP overall structure and the modifications occurring on the serine moiety were proved to exert little influence on their anti-QS activities, thus enlightening us on how to efficiently make derivatization of these series of CDPs to get better bioactivity. This work not only disclosed the progress of these tryptophane-containing cyclic dipeptides working on bacteriostatic and biocompatibility but also showed the right way to carry out the molecule design to improve the anti-virulence ability of this kind of compounds for the treatment of infectious disease. The mechanism of these CDPs on the QS system in *P. aeruginosa* would be further investigated in our following work. We anticipate that these biocompatible CDPs would be promising to be used as anti-virulence candidates against troublesome multi-drug resistance *P. aeruginosa*.

## 4. Materials and Methods

### 4.1. Materials and Synthetic Methods

All commercial organic solvents and salts (Sigma Aldrich, TCI, Adamas, J&K, Energy, etc.) were used without further purification unless otherwise stated. Cyclo(*L*-Trp-*L*-Ser) was synthesized using Boc-Trp-OH and *L*-Serine methyl ester hydrochloride as starting materials via a three-step synthetic procedure, as the literature described [36]. Briefly, Boc-Trp-OH and L-Serine methyl ester were coupled forming the amide bond in the presence of EDC•HCl and HOBt as the coupling reagents and Et_3_N as the base. After purification, the linear dipeptide was treated with TFA/dichloromethane (1:1) to remove the Boc group. Subsequently, the crude product was dissolved in MeOH and treated with ammonium hydroxide for cyclization. The product for bioactivity assay was purified using silica gel and prep-HPLC. The other CDPs were synthesized by GL Biochem (Shanghai) Ltd. (Shanghai, China), following the reported procedures [36,37], and the quality and purity were confirmed by NMR (Bruker 400 MHz) and HPLC (SHIMADZU LC-20A). The characterization data was illustrated in the Supporting Information.

Agar, glucose, and other salts for preparation of the mediums were purchased from Sinopharm Chemical Reagent Co., Ltd. (Shanghai, China) and Aladdin. Kanamycin sulfate was purchased from Sangon Biotech (Shanghai, China). Defibrinated Sheep Blood was purchased from Dening Bio. The human lung epithelial cell line A549 (ATCC CCL-185) was kindly donated by Professor Cuixia Chen, China University of Petroleum (East China). The mouse embryonic fibroblasts NIH-3T3 were donated by Dr. Hanping Fu, Fujian Normal University.

### 4.2. Strains and Growth Medium

*C. violaceum* CV026 (mini-Tn5 mutant of *C. violaceum* ATCC 31532) and *P. aeruginosa* PAO1 (ATCC 27853) strains were used in this study for anti-QS evaluation. *C. violaceum* CV026 was incubated in Luria-Bertani (LB) broth (yeast extract, 0.5%, *w*/*v*, peptone, 1%, *w*/*v*, NaCl, 1%, *w*/*v*) overnight at 28 °C under 150 rpm. Meanwhile, *P. aeruginosa* PAO1 was incubated in LB medium overnight at 37 °C under 165 rpm. The bacterial concentrations were determined using a microplate reader (BioTek, Winooski, VT, USA), and the bacteria were then diluted to the appropriate concentration for further mixing with tested CDPs.

### 4.3. Antimicrobial Assay and Bacterial Growth Measurement

These CDPs’ minimum inhibitory concentrations (MICs) were determined using a modified broth microdilution method, according to the Clinical and Laboratory Standards Institute (CLSI, 2012) [38]. Briefly, overnight activated *P. aeruginosa* PAO1 and *C. violaceum* CV026 cells were inoculated into Mueller-Hinton Broth or M63 Broth in the presence of different CDPs at a series of concentrations in 96-well microtiter plates. After incubation at 37 °C or 28 °C for 24 h, respectively, OD_600_ was tested to determine the bacterial density. The MICs were the lowest concentrations of the samples with visible cell growth inhibition.

The growth curves of *P. aeruginosa* PAO1 under treatment with different CDPs (at a concentration of 1 mM) were measured using a microplate reader. The bacterial cultures were diluted with LB medium to 10^7^ CFU/mL and incubated statically in 96-well microplates in the presence of each CDP. DMSO (1%, *v*/*v*) was used as the negative control. The bacterial density of each well was measured every 2 h for 24 h at a wavelength of 600 nm.

### 4.4. Violacein Quantification

The production of violacein of *C. violaceum* CV026 was measured to evaluate the potential anti-QS activity of these CDPs [39]. Overnight cultured *C. violaceum* CV026 was diluted with 20 mL LB medium to 10^7^ CFU/mL, followed by adding 20 μL kanamycin sulfate (85 mM) and 100 μL of C_6_HSL (2 mM), then 2 mL of the above bacterial solution was added into a 24-well plate with different cyclic dipeptides at a final concentration of 1 mM. The mixtures were allowed to shake at 150 rpm at 28 °C for 12 h. One milliliter of the overnight-grown cell culture was transferred to a 1.5 mL Eppendorf tube and centrifuged at 12,000 rpm for 10 min. The supernatant was discarded, and the sediments were subsequently dissolved with 1 mL DMSO. After centrifugation at 12,000 rpm for 10 min, the obtained supernatant was added into a 96-well plate, and the absorbance was measured at a wavelength of 585 nm via a microplate reader. The percent changes based on the control group in absorption intensity were calculated to quantify the decrease of violacein production.

### 4.5. Docking

The energies of the CDPs were minimized with ChemBio3D Ultra 14.0, and the structures were saved as PDB form. The structure of autoinducer C_6_HSL was downloaded from ZINC. The crystal structure of *C. violaceum* CviR (PDB ID: 3QP1, resolution of 2.0 Å) was downloaded from the RCSB PDB website. Before docking, the protein’s co-crystal organic moiety and H_2_O molecules were removed, and polar hydrogens were added to the protein. Docking was accomplished using AutoDockTools-1.5.6 (downloaded from http://mgltools.scripps.edu/, on 3 December 2020), following a general protocol [19]. The grid box was set to the whole receptor involved in the active site. Parameters were set to a Lamarckian genetic algorithm (GA) calculation of 100 runs. The resulting docked poses were analyzed using cluster analysis and ranked by binding energy. PyMOL was used for visualization.

### 4.6. Pyocyanin Quantification and Elastase Activity Assay

Pyocyanin production was measured as previously reported [18]. Briefly, the overnight cultured *P. aeruginosa* PAO1 cultures were diluted with LB media to 10^7^ CFU/mL, then incubated statically in 24-well microplates with different CDP concentrations at 37 °C for 18 h. DMSO (1%, *v*/*v*) was served as the negative control. After incubation, the cultures were centrifuged at 12,000 rpm for 10 min. The supernatants were extracted with chloroform, followed by pre-extraction with 0.2 M HCl. The solution absorbance was measured at a wavelength of 520 nm. The percent changes in absorption intensity were calculated to determine the inhibitory capacity of these CDPs against pyocyanin production.

Elastase activity was investigated using an elastin-Congo-red assay [19]. *P. aeruginosa* PAO1 cultures were treated as described above in pyocyanin assay. After centrifugation, the supernatant was filtered using a 0.22 μm nylon filter. To a clean tube, 100 μL of filtered solution was added and 400 μL Tris-HCl (pH = 7.5) containing 4 mg elastin-congo red (Bomei, Dongguan, China). The mixture was shaken at 37 °C for 10 h, followed by centrifugation at 11,000× *g* for 10 min. The absorbance of the supernatant was measured spectrophotometrically at 495 nm. All experiments were performed in triplicates.

### 4.7. Swimming Motility

The swimming motility of *P. aeruginosa* PAO1 was assayed using a published method [40,41]. Briefly, each CDP solution (100 mM in 20 μL) was diluted with 2 mL molten swim agar (pH 7.2), which consisted of peptone (1%, *w*/*v*), sodium chloride (0.5%, *w*/*v*), and agar (0.3%, *w*/*v*), to a final concentration of 1 mM. The cultures were dispensed onto sterile Petri dishes and allowed to solidify at room temperature for 30 min. A 2 μL 10^7^ CFU/mL PAO1 bacterial culture was inoculated with a pipette into the center of a 35 mm swimming agar plate and then cultured at 37 °C for 18 h. DMSO (1%, *v*/*v*) was used as the negative control. The swimming zones were measured after incubation at 37 °C for 18 h.

### 4.8. QS Genes Expression Assay

Expressions of *P. aeruginosa* PAO1 QS genes were investigated following the reported protocol [42]. The overnight cultured bacterial cells were inoculated on 2 mL fresh LB medium containing different CDPs (1 mM) in 6-well microplates, regulating the final bacterial density at 10^7^ CFU/mL. After incubation at 37 °C for 10 h, the majority of QS genes were maximally regulated, and the bacterial cells were collected through centrifugation at 4 °C at 8000 rpm. The cells (1 mL) were treated with RNAiso Plus, and chloroform (200 µL) was subsequently added to each sample. The tubes were incubated for 5 min at room temperature and centrifuged at 12,000 rpm for 15 min at 4 °C. Afterward, the upper colorless aqueous layer (400 μL) was collected in a new RNAase-free Eppendorf tube. Isopropanol (500 µL) was added to each tube, and the tubes were vortexed and stood for 10 min, then centrifuged at 12,000 rpm for 10 min at 4 °C. After discarding the supernatant, the RNA pellet was washed twice with 75% ethanol (1 mL) and air-dried for 10–15 min. Reverse transcription was accomplished with the iScript^TM^ cDNA Synthesis kit (TaKaRa Bio, Kusatsu, Japan). The RT-qPCR reaction mixture was composed of 2 μL cDNA, 10 µL iTaq^TM^ Uiversal SYBR Green supermix (2x), 2 μL each primer (Table S1), and 6 μL RNAase free water. The reaction was then performed on Bio-Rad Laboratories (Hercules, CA, USA). The total reaction volume was 20 μL. Cycle conditions were as follows: 95 °C for 3 min followed by 40 cycles of 95 °C for 3 s and 60 °C for 30 s. The ribosomal gene, *rpo*D, was chosen as a control to normalize the RT-PCR data and calculate the relative gene expression changes with the (ΔΔCq) method.

### 4.9. Biofilm Assay

#### 4.9.1. Inhibition of Biofilm Formation

The biofilm formation assay was conducted using a crystal violet stain method, as previously reported [43]. The overnight cultured *P. aeruginosa* PAO1 was diluted with LB medium to 10^7^ CFU/mL and incubated statically in 96-well plates in the presence of different CDPs or DMSO (1%, *v*/*v*). After incubation at 37 °C for 24 h, the plates were carefully washed three times with ultrapure water to remove the cultures. The remaining biofilms were dried at 50 ℃ for 30 min and stained with 0.5% crystal violet for 30 min at room temperature. The plates were then washed three times with ultrapure water to remove the excess dye. The attached staining was dissolved in 75% ethanol for 30 min, and OD_595_ was detected by a microplate reader. Each test was performed independently in triplicates.

#### 4.9.2. Biofilm Dispersion Assay

The biofilm dispersion was assayed following the previous procedure [44]. Briefly, *P. aeruginosa* PAO1 cultures (10^7^ CFU/mL) were added to a 96-well plate, 100 μL per well, and incubated at 37 °C for 48 h. The planktonic cell cultures were then removed from the plate and washed three times with PBS. Fresh LB media containing different CDPs was added to each well, and DMSO (1%, *v*/*v*) was used as the control group. After incubation for 6 h, the crystal violet stain method was conducted as described in Section 4.9.1.

#### 4.9.3. Observation of Morphology

Morphologies of the bacteria and bacterial biofilm were observed using scanning electron microscopy (SEM). The SEM samples were prepared as previously reported [45]. The *P. aeruginosa* PAO1 bacterial suspension (10^7^ CFU/mL) was incubated in the presence or absence of tested CDPs (1 mM) in 6-well microplates with a piece of glass on each bottom at 37 °C for 48 h. Subsequently, the coverslips were washed with PBS, and the residual bacterial biofilm was fixed with 2.5% glutaraldehyde for 12 h. After removing the remaining glutaraldehyde, dehydration of the samples was performed with gradient concentrations of ethanol (50%, 70%, 80%, 90%, 100%, *v*/*v*). At last, the samples were airdried and sputtered with gold.

### 4.10. Adherence Assay

The anti-adhesion ability of these CDPs against *P. aeruginosa* PAO1 onto the PVC surface was evaluated using the plate counting method [46,47]. *P. aeruginosa* PAO1 cultures (10^7^ CFU/mL) were added to a 96-well plate, 100 μL per well, and cultured with or without CDPs (1 mM) at 37 °C for 3 h. After incubation, the planktonic cells and culture medium were rinsed out using PBS. 100 μL PBS was added to each well, and the plate was sonicated at 40 Hz for 30 min at room temperature. The contents of each well were diluted 10 times with 0.9% NaCl solution, and 100 μL of the dilutions were spread onto LB agar plates. After incubation at 37 °C for 18 h, the colonies on plates were counted using Image J.

### 4.11. Cytotoxicity and Hemolytic Activity

Cytotoxicity assay was conducted through the MTT method [48]. Briefly, A549 and NIH-3T3 were proliferated in F-12K or Dulbecco’s Modified Eagle’s Medium (DMEM) within 1% Penicillin-streptomycin and 10% heat-inactivated fetal bovine serum under 5% CO_2_ at 37 °C. A549 or NIH-3T3 cells (2000 cells per well) were seeded into a 96-well plate and incubated at 37 °C for 24 h under a 5% CO_2_ atmosphere. The cells were then treated with CDPs in different concentrations ranging from 0.1 to 10 mM, and PBS (1% DMSO)-treated cells were used as the control group. All cells were cultured for 24 h under the same cultivating conditions. Subsequently, the attached cells were washed twice with the medium. A total of 10 μL of PBS containing MTT (5 mg/mL) was added to each well and co-cultured with the cells for a further 4 h. The culture medium was then removed, and DMSO was added and co-incubated at 37 °C for 20 min on a shaker at 120 rpm to dissolve the generated formazan crystal. The absorbance of each well was measured using a microplate reader at a wavelength of 570 nm. The cytotoxicity of the CDPs was evaluated using the relative viability of the cell, for which the blank control was 100%. Every tested concentration was repeated independently three times.

The hemolytic activities of these cyclic dipeptides were evaluated using sheep red blood cells (sRBCs). Briefly, sRBCs were suspended in PBS buffer (pH 7.4) at 2% (*v*/*v*) and mixed thoroughly with CDPs at different concentrations. After incubation at 37 °C for 4 h, the cells were centrifugated at 7100× *g* for 1 min, and the supernatant was collected and added into a 96-well plate. The hemoglobin release was measured at 540 nm using a microplate reader. PBS (1% DMSO) and 0.1% Triton X-100 were used as negative and positive controls. Each test was performed three times independently. The hemolytic activity of each CDP can be calculated using the following equation:

$$\mathrm{Hemolytic}\;\mathrm{activity}\;(\%)=\;\frac{A540_{\;CDP\;}-A540_{PBS}}{A540_{\mathrm{Triton}\;X-100\;}-A540_{PBS}}\times 100\%$$

## Supplementary Materials

The following are available online at https://www.mdpi.com/1660-3397/20/2/85/s1, Figure S1: Docked conformation of the natural ligand and CDPs with CviR; Figure S2: Effects of synthetic CDPs over a range of concentrations (0.1–1 mM) on pyocyanin production of *P. aeruginosa* PAO1.; Figure S3: Effects of synthetic CDPs over a range of concentrations (0.1–1 mM) on elastase production of *P. aeruginosa* PAO1; Figure S4: Effects of synthetic CDPs over a range of concentrations (0.1–1 mM) on biofilm production of *P. aeruginosa* PAO1; Table S1: Specific amplification primer sets for PAO1; Scheme S1: Synthesis of c(WS); Table S2: The FT-IR data of CDPs; Figure S5: Circular dichroism spectra of c(WS), c(W*s*), c(*w*S), c(*ws*); Figures S6–S21: The NMR spectrum of CDPs; Figures S22–S29: The HPLC analysis of CDPs.
