# Supplementary material for: Anti-Quorum-Sensing Activity of Tryptophan-Containing Cyclic Dipeptides
Source: Mar Drugs. 2022 Jan 19;20(2):85. doi: 10.3390/md20020085 (PMC8924889; doi:10.3390/md20020085)

Supporting Information

Anti-Quorum-Sensing Activity of Tryptophan-containing Cyclic Dipeptides

Yinglu Wang†, Qian Zheng†, Li Li, Lile Pan and Hu Zhu*

Engineering Research Center of Industrial Biocatalysis, Fujian Provincial Key Laboratory of Advanced Materials Oriented Chemical Engineering, Fujian Provincial Key Laboratory of Polymer Materials, College of Chemistry and Materials Science, Fujian Normal University, Fuzhou 350007, China. wangyl@fjnu.edu.cn (Y.W.); qsxzhengq@163.com (Q.Z); lilifnju@163.com (L.L); panlljxgz@163.com (L.P.).
* Correspondence: zhuhu@fjnu.edu.cn (H.Z.)
† These authors contribute equally.

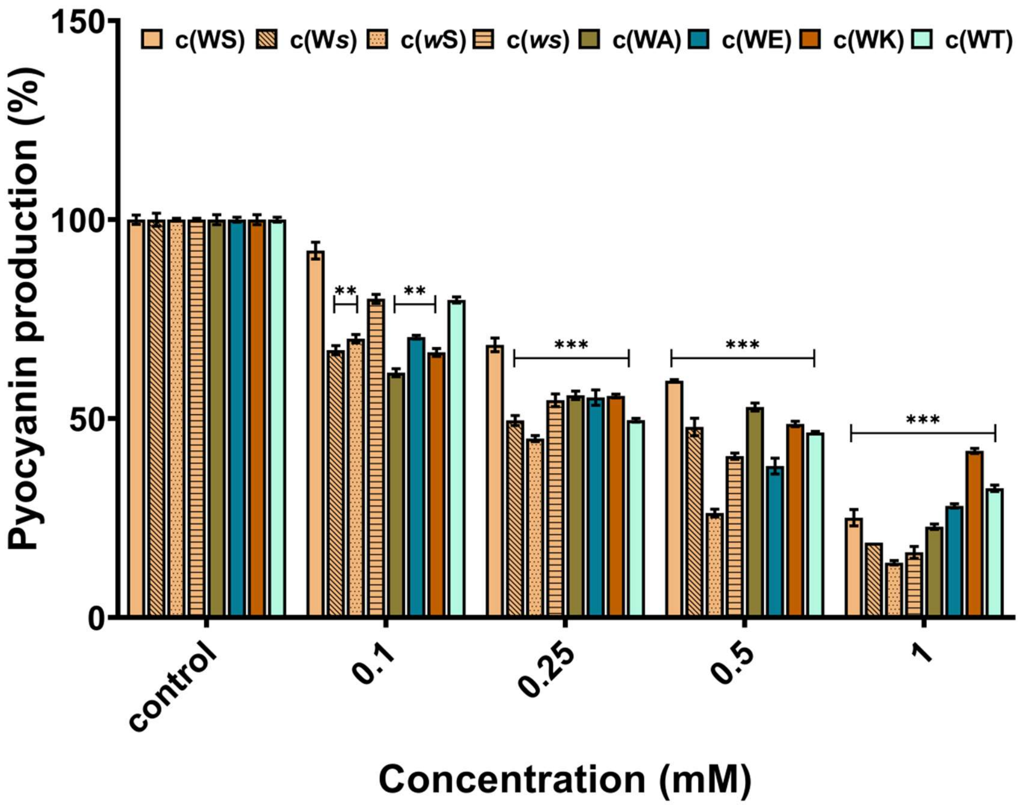

Figure S1. Effects of eight synthetic CDPs over a broad range of concentrations (0.1-1 mM) on pyocyanin production of *P. aeruginosa* PAO1. Data are expressed as the percentage of residual pyocyanin after 18 h of treatment compared with the untreated sample. Each performed at least in triplicate. Differences in mean absorbance were compared to the untreated control and considered significant when p* < 0.05, p** < 0.01, p*** < 0.001 according to the ANVOA analysis.

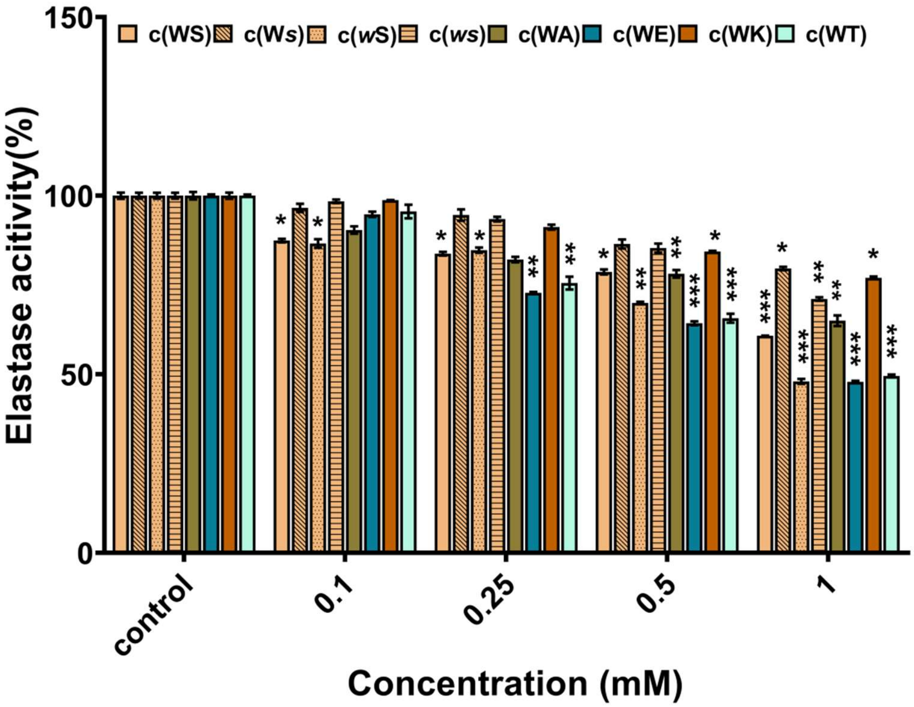

Figure S2. Effects of eight synthetic CDPs over a broad range of concentrations (0.1-1 mM) on elastase production of *P. aeruginosa* PAO1. DMSO (1%, v/v) was served as the control. Data are expressed as the percentage of residual elastase after 18 h of treatment compared with the untreated sample. Each performed at least in triplicate. Differences in mean absorbance were compared to the untreated control and considered significant when p* < 0.05, p** < 0.01, p*** < 0.001 according to the ANVOA analysis.

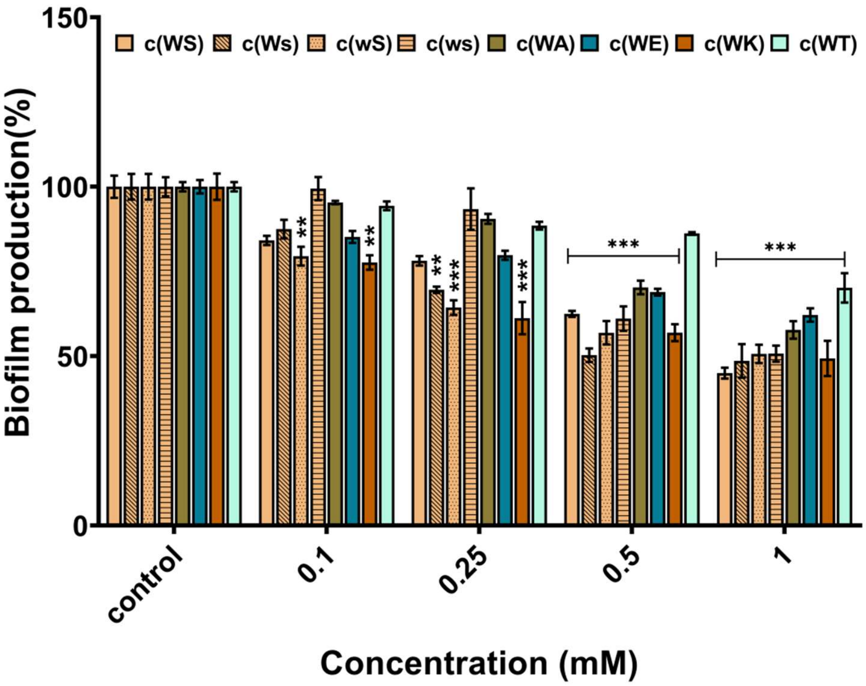

Figure S3. Effects of eight synthetic CDPs over a broad range of concentrations (0.1-1 mM) on biofilm production of *P. aeruginosa* PAO1. DMSO (1%, v/v) was served as the control. Data are expressed as the percentage of residual biofilm after 24 h of treatment compared with the untreated sample. Each performed at least in triplicate. Differences in mean absorbance were compared to the untreated control and considered significant when p* < 0.05, p** < 0.01, p*** < 0.001 according to the ANVOA analysis.

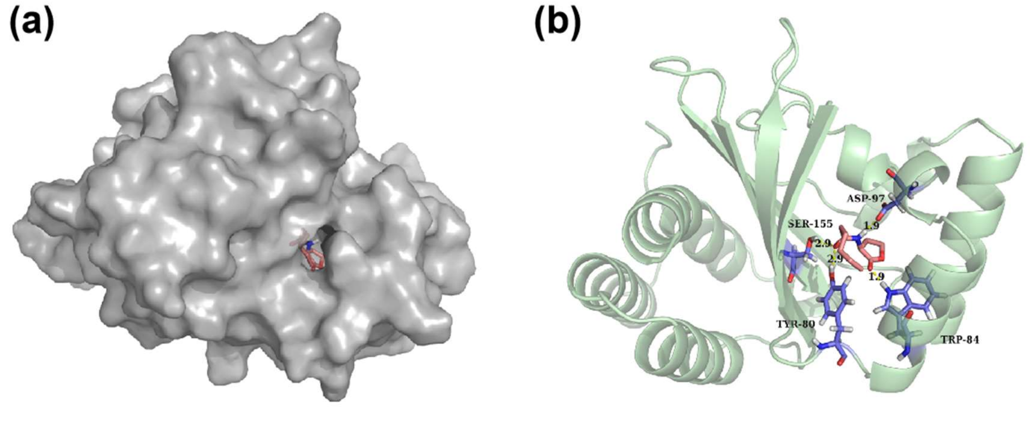

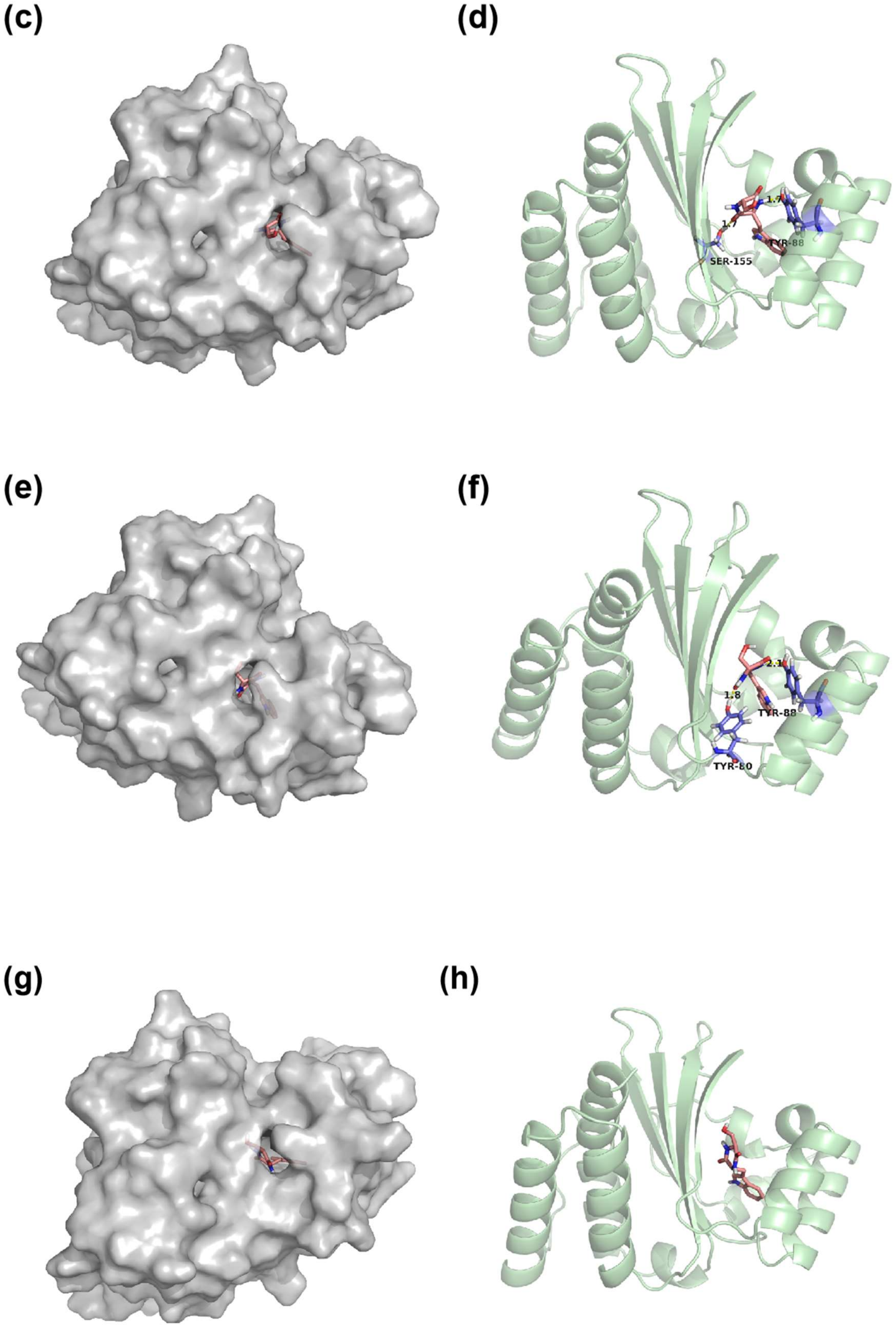
(c)
(d)
1.7
1.7
SER-155
TYR-88
(e)
(f)
2.1
1.8
TYR-88
TYR-80
(g)
(h)

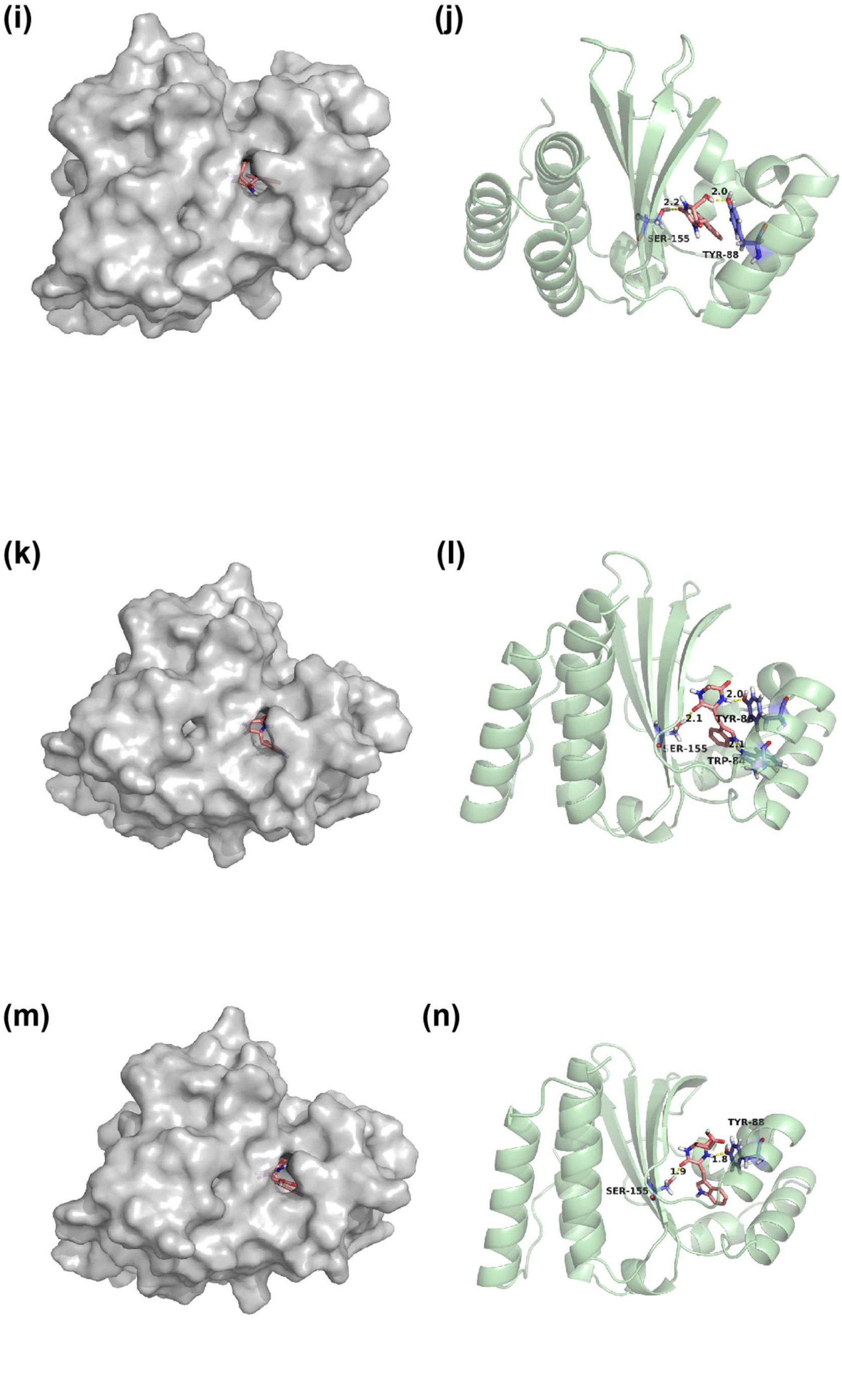

(i)
(j)
2.2
2.0
SER-155
TYR-88
(k)
(l)
2.0
2.1
TYR-88
SER-155
2.1
TRP-84
(m)
(n)
TYR-88
1.9
1.8
SER-155

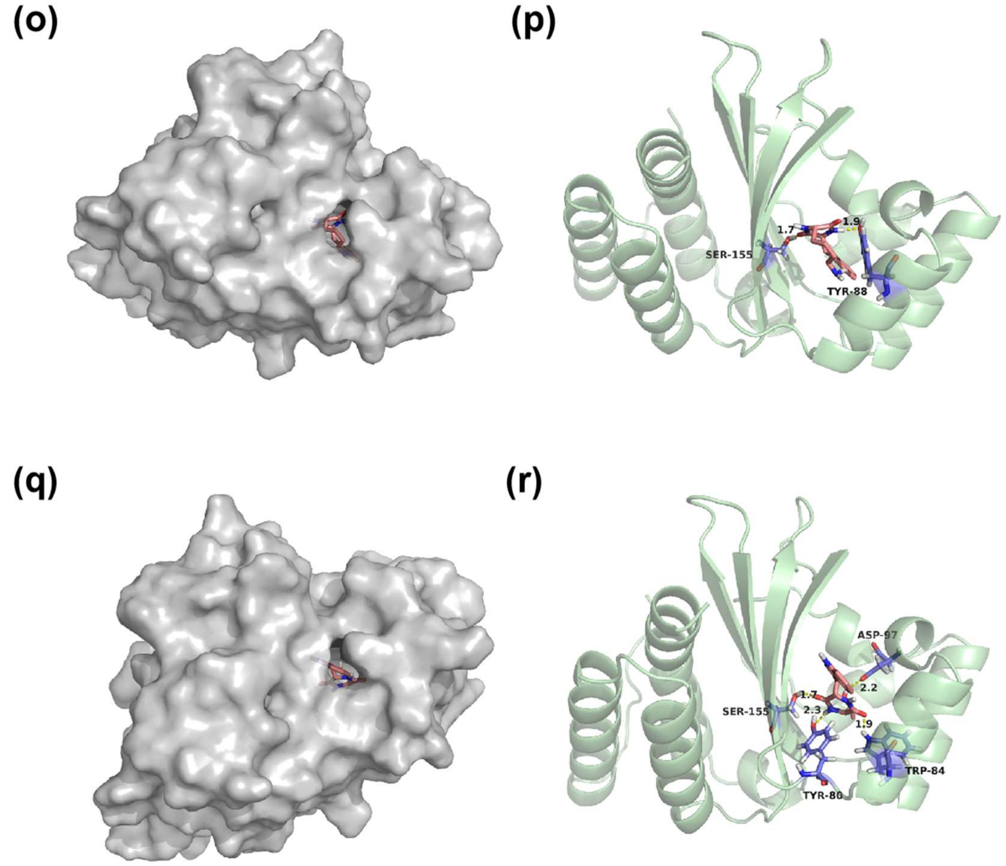

Figure S4. Docked conformation of natural ligand **C_6-HSL** (a, b) and (c, d): **c(WS)**, (e, f): **c(Ws)**, (g, h): **c(wS)**, (i, j): **c(ws)**, (k,l): **c(WA)**, (m, n): **c(WE)**, (o, p): **c(WK)**, (q, r): **c(WT)** with CviR protein.

Table S1. Specific amplification primer sets for PAO1

Gene name	Primer type	Primer Sequence（5’-3’）
lasI	Fw	CGTGCTCAAGTGTTCAAGGA
	Rev	GCGTCTGGATGTCGTTCTG
lasR	Fw	CTGTGGATGCTCAAGGACTAC
	Rev	ACCGAACTTCCGCCGATT
rhlI	Fw	GCTACATCGTCGCCATGAG
	Rev	TCTCGCCCTTGACCTTCTG
rhlR	Fw	CCGATGCTGATGTCCAACC
	Rev	GGAAGTTCACCGTGCTCTC
pqsA	Fw	GACCGGCTGTATTCGATTC
	Rev	GCTGAACCAGGGAAAGAAC
pqsR	Fw	CTGATCTGCCGGTAATTGG
	Rev	ATCGACGAGGAACTGAAGA
rpoD	Fw	CGAACTGCTTGCCGACTT
	Rev	GCGAGAGCCTCAAGGATAC

Scheme S1 Synthesis of c(WS)[1] and its isomers

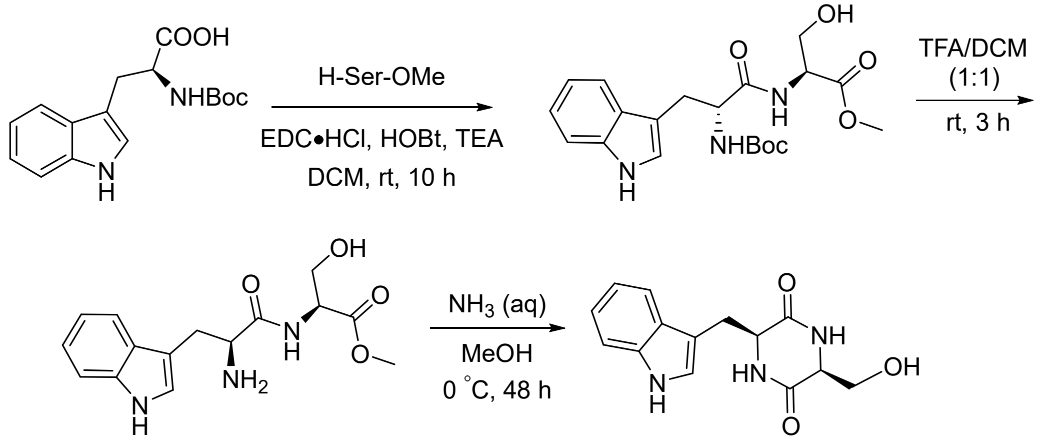

Boc-L-Trp (5.00 g, 16.45 mmol), H-Ser-OMe•HCl (3.82 g, 24.67 mmol), EDC•HCl (4.74 g, 24.67 mmol) and HOBt (3.33 g, 24.67 mmol) were dissolved in 45 mL anhydrous DCM. Then, Et_3N (10.30 mL, 74.03 mmol) was added to the mixture dropwise at 0 °C. The reaction was allowed to stir at room temperature 10 h, then the mixture was filtered through Celite. The filtrate was washed with a saturated solution of NH_4Cl (×3) and $NaHCO_3$ (×3), and dried over $MgSO_4$. Purification using silica gel column chromatography (PE/EA = 1:2) afforded the protected dipeptide as a white powder.

[1] Tian, W.; Sun, C.; Zheng, M.; Harmer, J. R.; Yu, M.; Zhang, Y.; Peng, H.; Zhu, D.; Deng, Z.; Chen, S. L.; Mobli, M.; Jia, X.; Qu, X., Efficient biosynthesis of heterodimeric C(3)-aryl pyrroloindoline alkaloids. *Nat Commun* **2018,** 9, 4428.

The obtained protected dipeptide was then dissolved in anhydrous DCM with 50% TFA solution, and was stirred at room temperature for 3 h. Then the resulting crude product was concentrated and dissolved in 70 mL MeOH and 35 mL ammonia (28 to 30% ammonium hydroxide in H_2O) at 0 °C. The reaction was allowed to stir at 0 °C for 48 h, and the resulting white precipitate was then collected. The product was purified using silica gel (MeOH/DCM = 1:10).

As enantiomers, c(WS) and c(*ws*) appeared the similar NMR data, which is consistent with literature[1]. NMR data of c(W*s*) and c(*w*S) are similar as:

^1H NMR (400 MHz, DMSO-d_6) δ 10.90 (s, 1H), 8.01 (s, 1H), 7.72 (s, 1H), 7.59 (d, *J* = 7.9 Hz, 1H), 7.31 (d, *J* = 8.1 Hz 1H), 7.17 (d, *J* = 2.3 Hz, 1H), 7.03 (dd, app.t, *J* = 7.4 Hz, 1H), 6.94 (dd, app.t, *J* = 7.5 Hz, 1H), 4.95 (t, *J* = 5.2 Hz, 1H), 4.11 (t, *J* = 3.8 Hz, 1H), 3.56 – 3.61 (m, 1H), 3.42-3.37 (m, 1H), 3.24 (dd, *J* = 14.5, 4.2 Hz, 1H), 3.10 (m, 1H), 3.03 (dd, *J* = 14.5, 4.5 Hz, 1H); ^{13}C NMR (100 MHz, DMSO-d_6) δ 168.18, 166.88, 135.96, 127.82, 124.62, 120.88, 118.98, 118.42, 111.26, 108.64, 62.56, 56.69, 55.11, 28.43.

FT-IR data of eight cyclic dipeptides

c(WS) IR (KBr): ν_{max} 3325.60, 1667.22, 1458.44, 1329.54, 745.80 cm^{-1}

c(Ws) IR (KBr): ν_{max} 3411.27, 1667.73, 1457.97, 1327.46, 745.94 cm^{-1}

c(wS) IR (KBr): ν_{max} 3330.33, 1669.96, 1458.73, 1331.56, 746.73 cm^{-1}

c(ws) IR (KBr): ν_{max} 3412.68, 1667.54, 1457.84, 1326.82, 746.12 cm^{-1}

c(WA) IR (KBr): ν_{max} 3414.85, 3190.27, 1659.29, 1459.11, 1325.95, 743.59 cm^{-1}

c(WE) IR (KBr): ν_{max} 3303.26, 3054.45, 1668.8, 1477.17, 1361.13, 742.33 cm^{-1}

c(WK) IR (KBr): ν_{max} 3365.55, 3188.83, 3046.57, 1671.14, 1459.7, 755.86 cm^{-1}

c(WT) IR (KBr): ν_{max} 3403.14, 3203.25, 3058.32, 1686.68, 1458.12, 749.14 cm^{-1}

Circular dichroism spectra of c(WS), c(Ws), c(wS), c(ws)

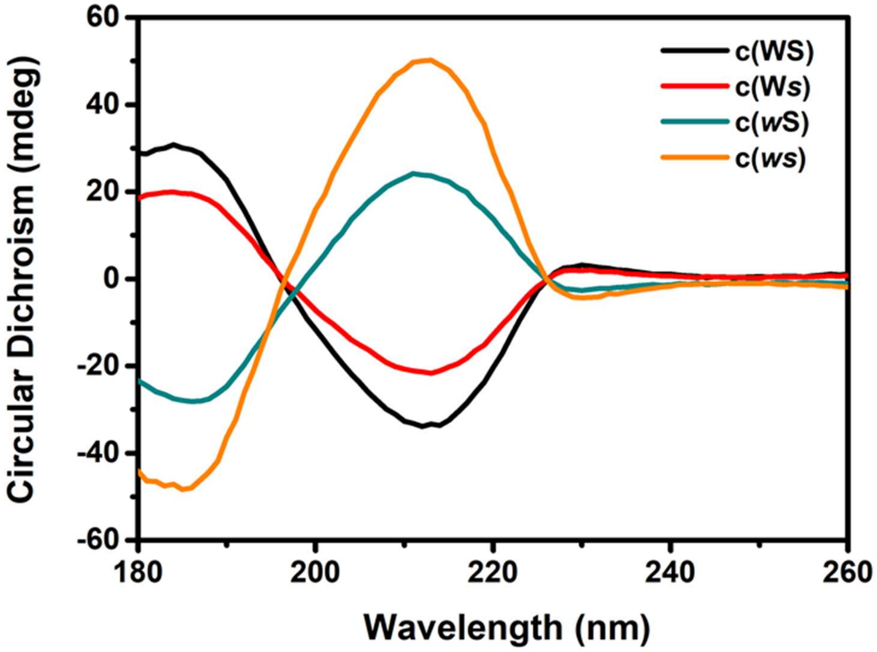

NMR Spectra. of cyclic dipeptides

c(WS) - ^{1}H NMR Spectrum - DMSO-d_6, 400 MHz

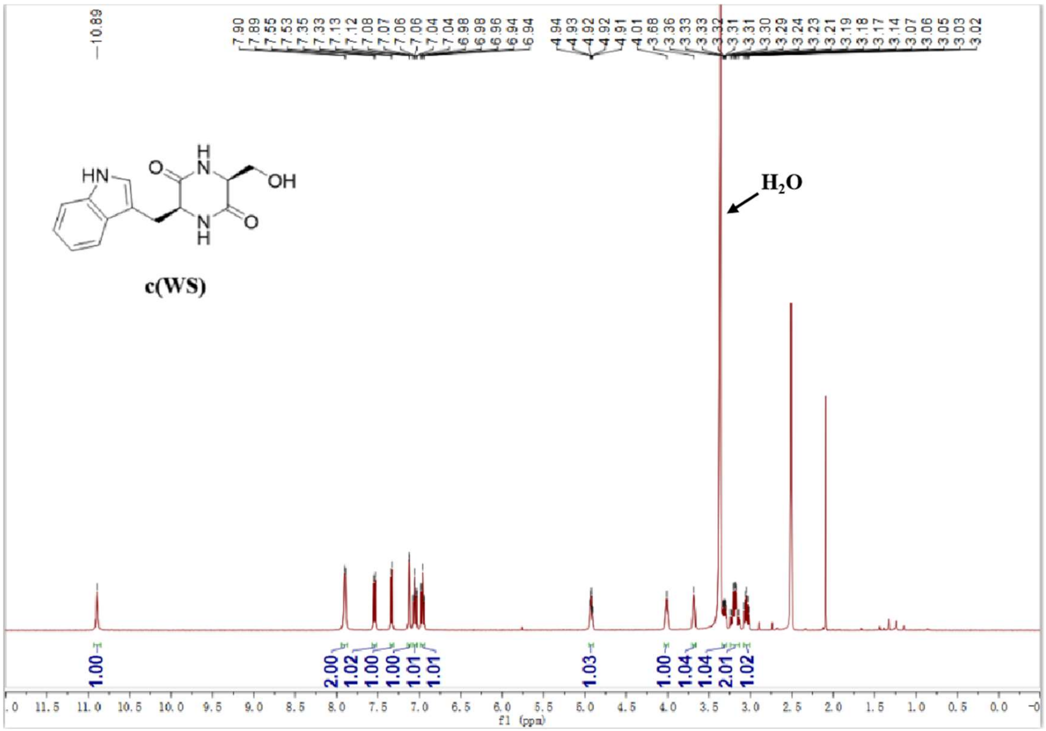

c(WS) – ^{13}C NMR Spectrum - DMSO-d_6, 100 MHz

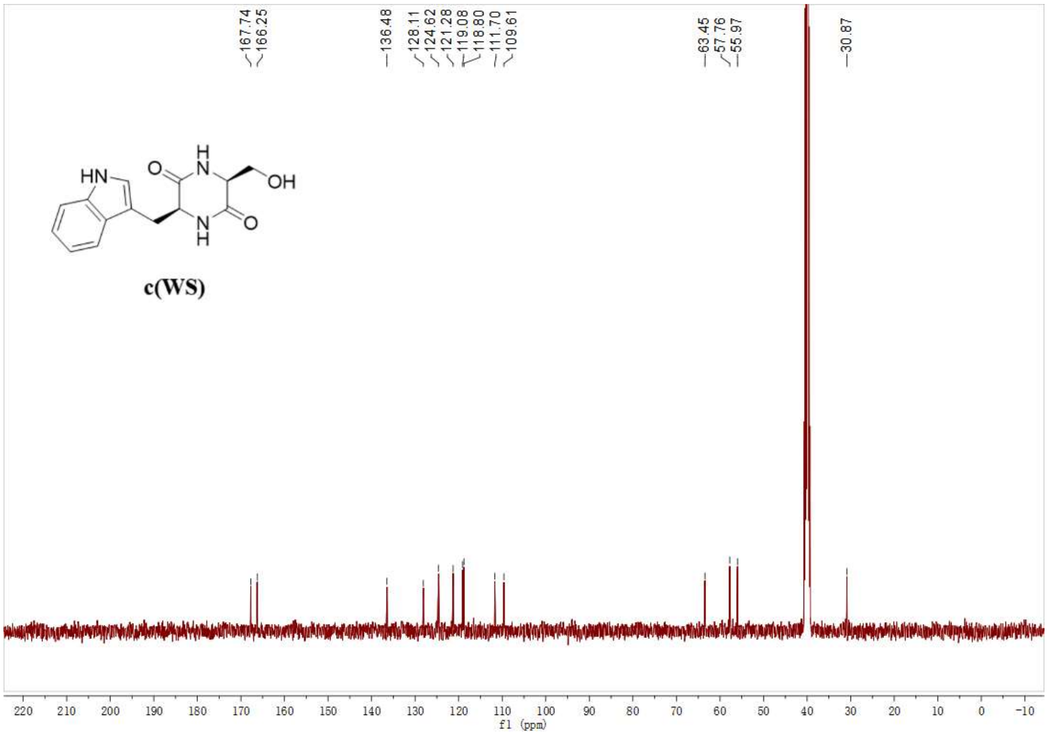

c(W*s*) - ^{1}H NMR Spectrum - DMSO-*d*$_6$, 400 MHz

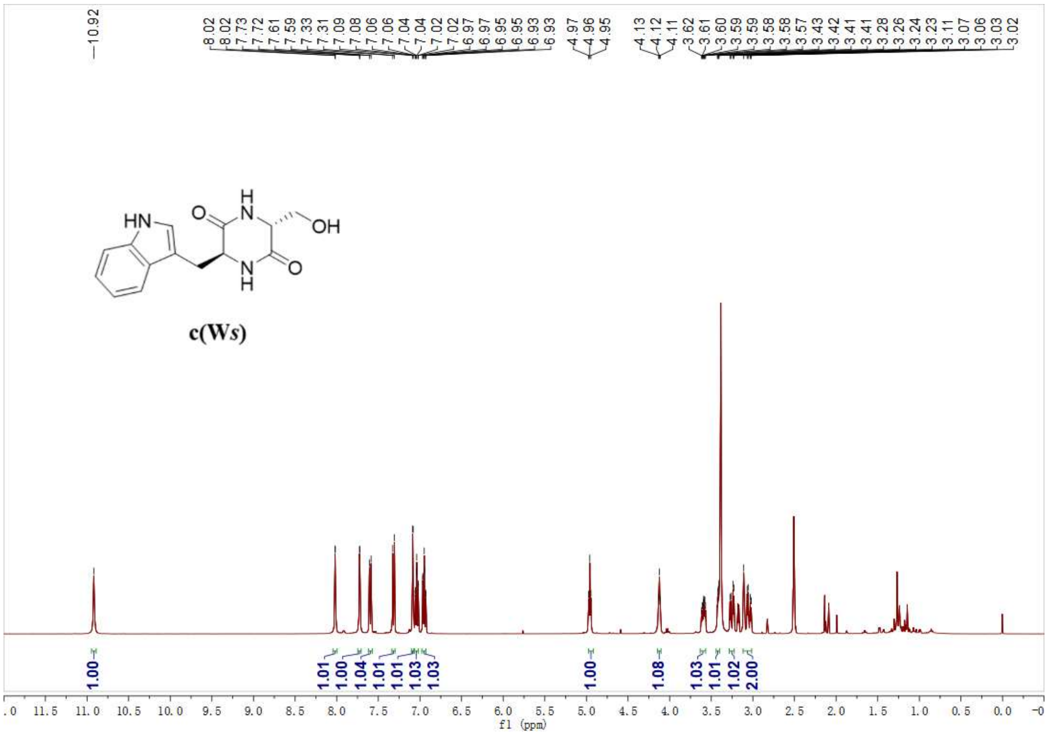

c(W*s*) – ^{13}C NMR Spectrum - DMSO-*d*$_6$, 100 MHz

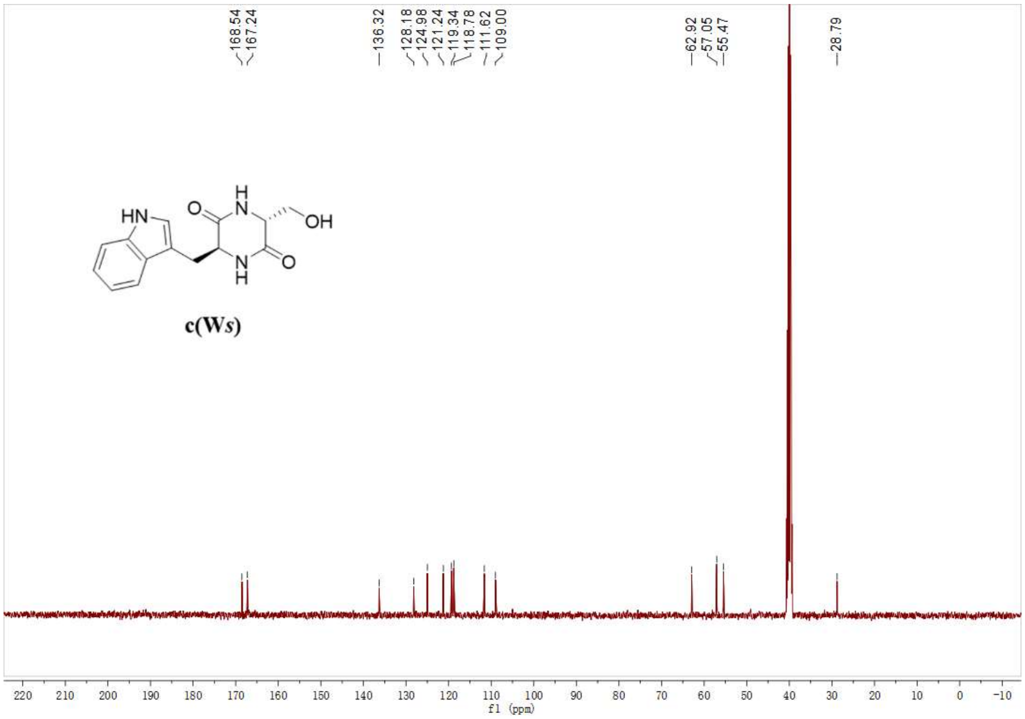

c(*w*S) - ^{1}H NMR Spectrum - DMSO-d_6, 400 MHz

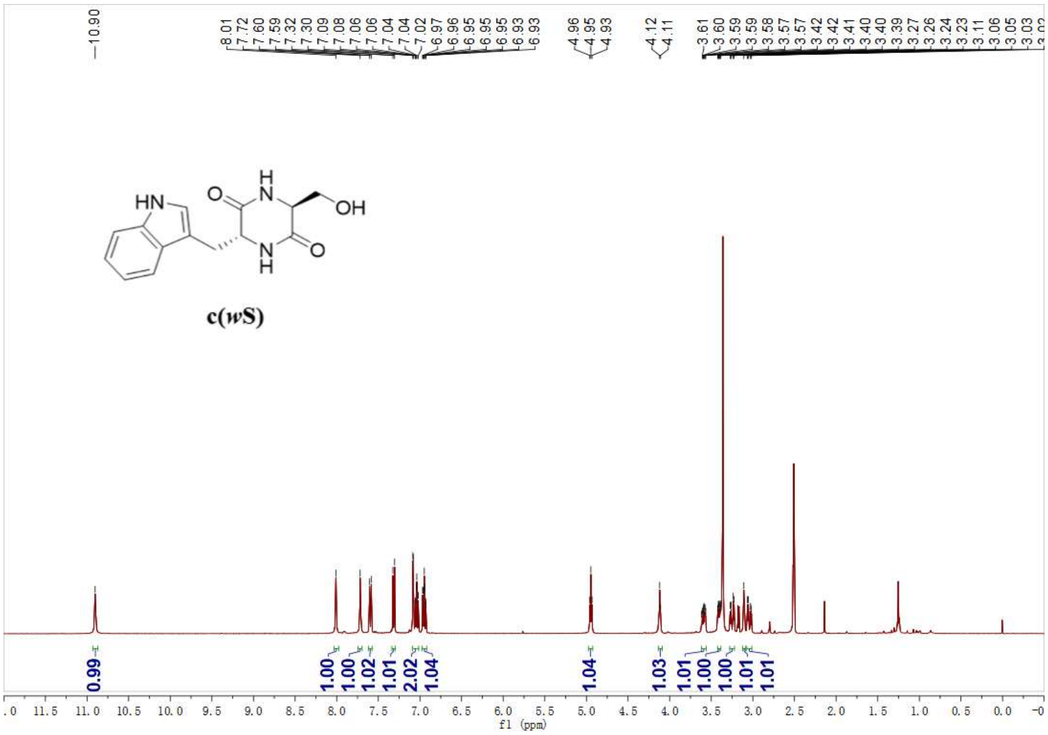

c(*w*S) – ^{13}C NMR Spectrum - DMSO-d_6, 100 MHz

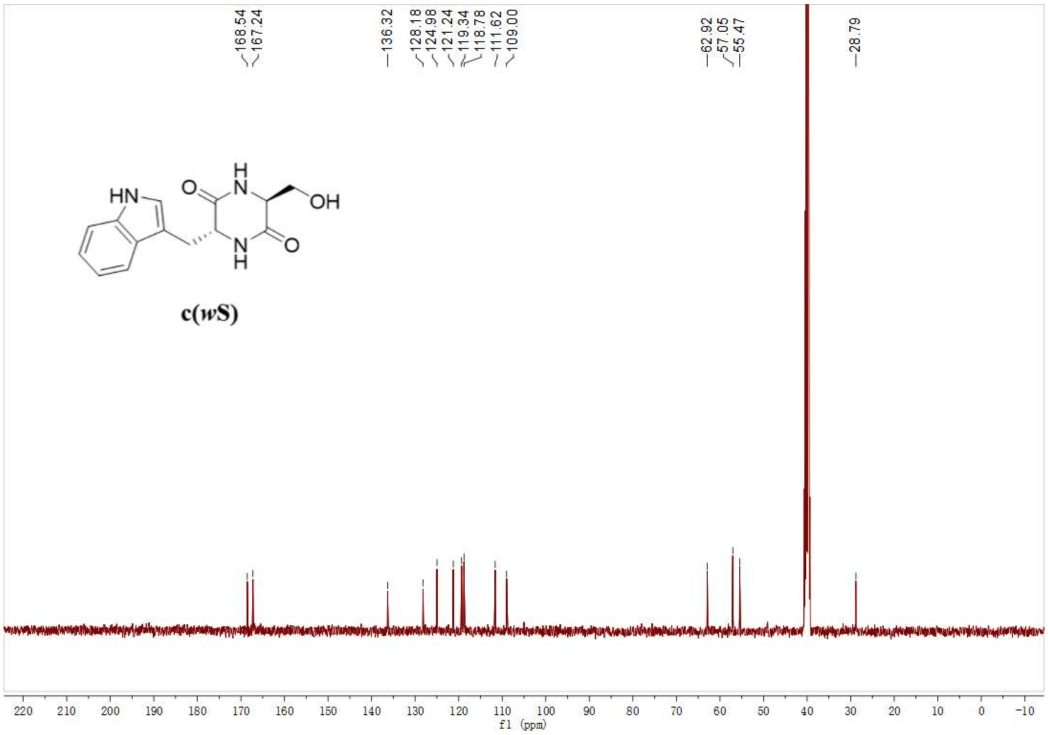

c(*w*s) - ^{1}H NMR Spectrum - DMSO-d_6, 400 MHz

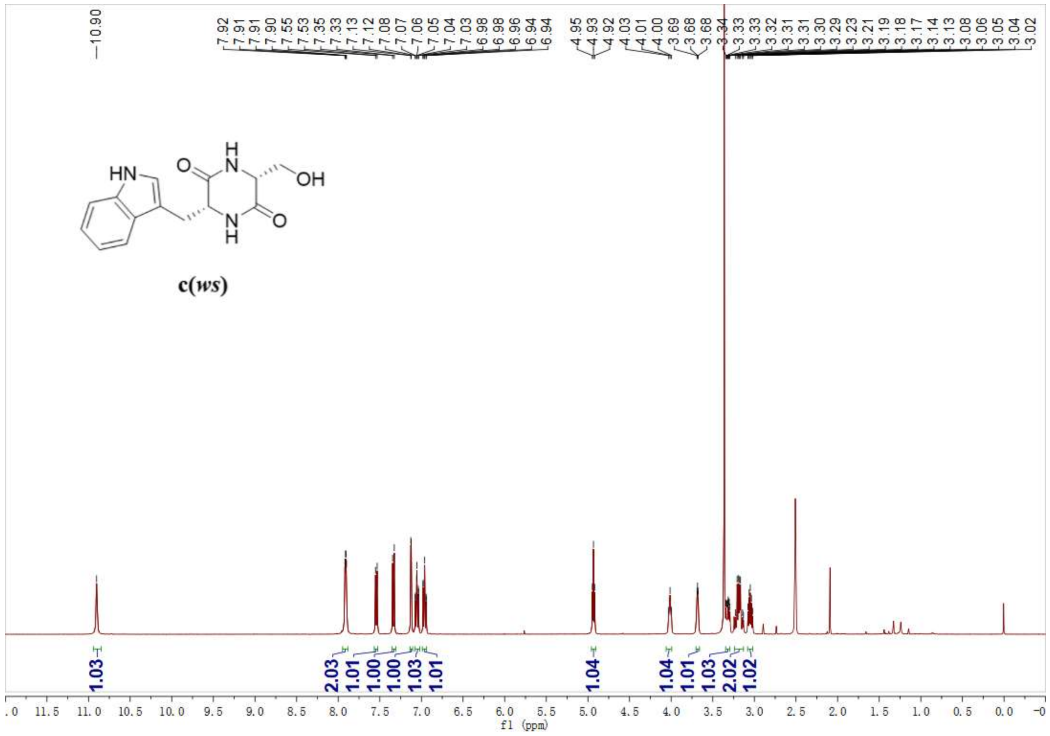

c(*w*s) – ^{13}C NMR Spectrum - DMSO-d_6, 100 MHz

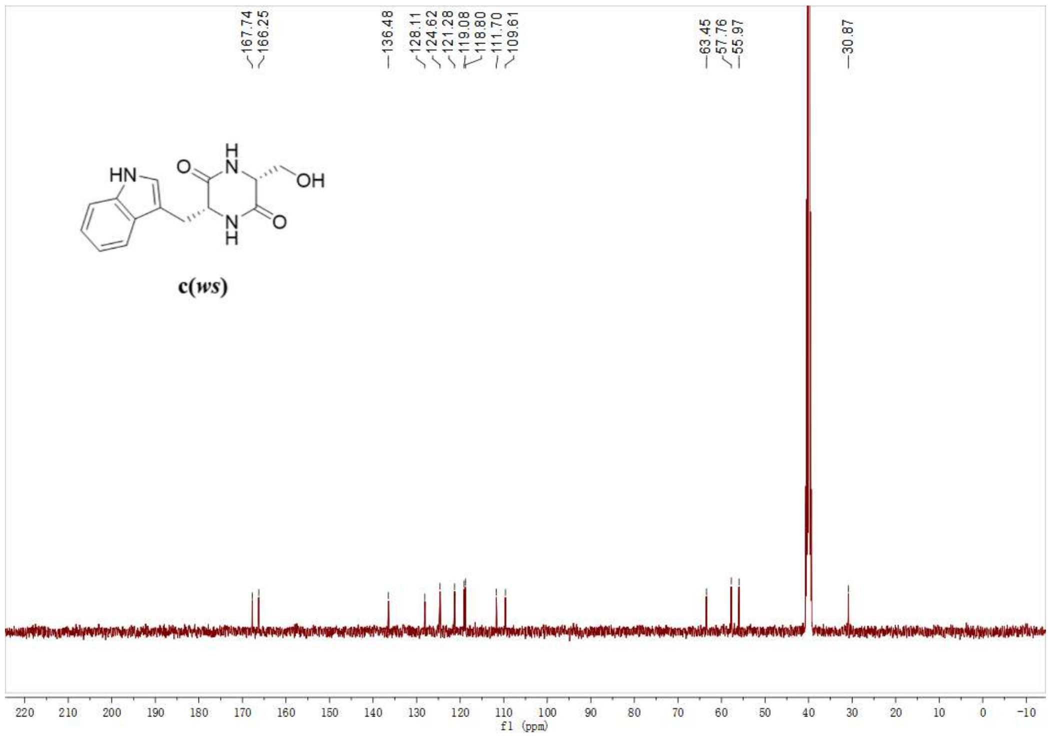

c(WA) - ^{1}H NMR Spectrum - DMSO-d_6, 400 MHz

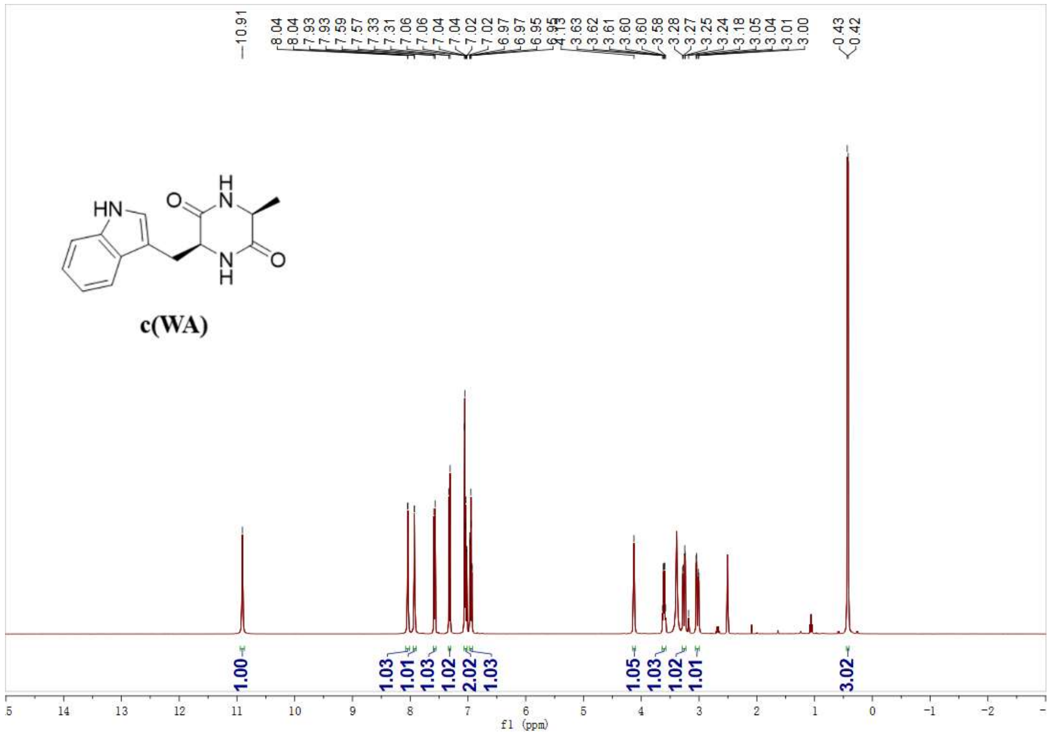

c(WA) – ^{13}C NMR Spectrum - DMSO-d_6, 100 MHz

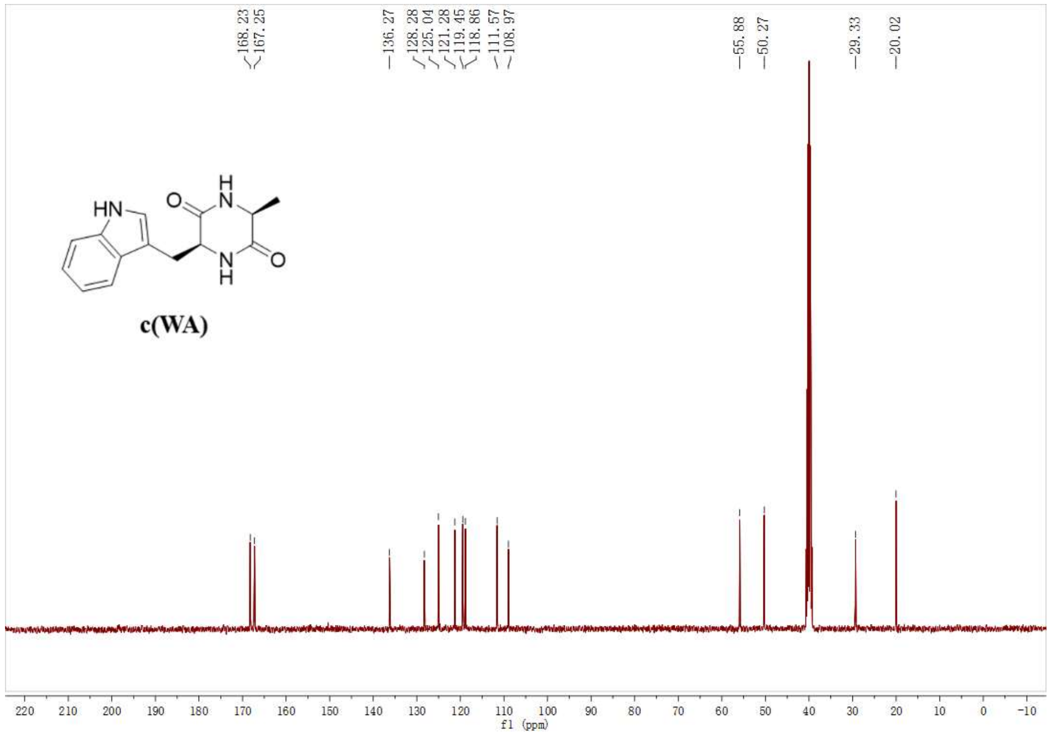

c(WT) - ^{1}H NMR Spectrum - DMSO-d_6, 400 MHz

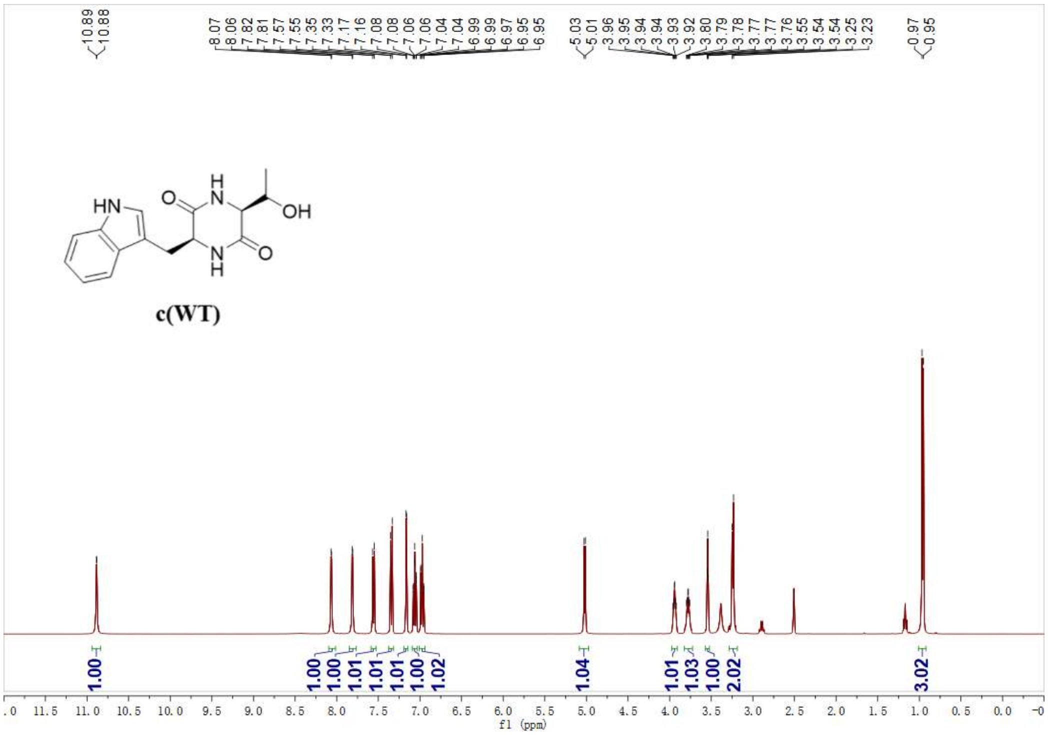

c(WT) – ^{13}C NMR Spectrum - DMSO-d_6, 100 MHz

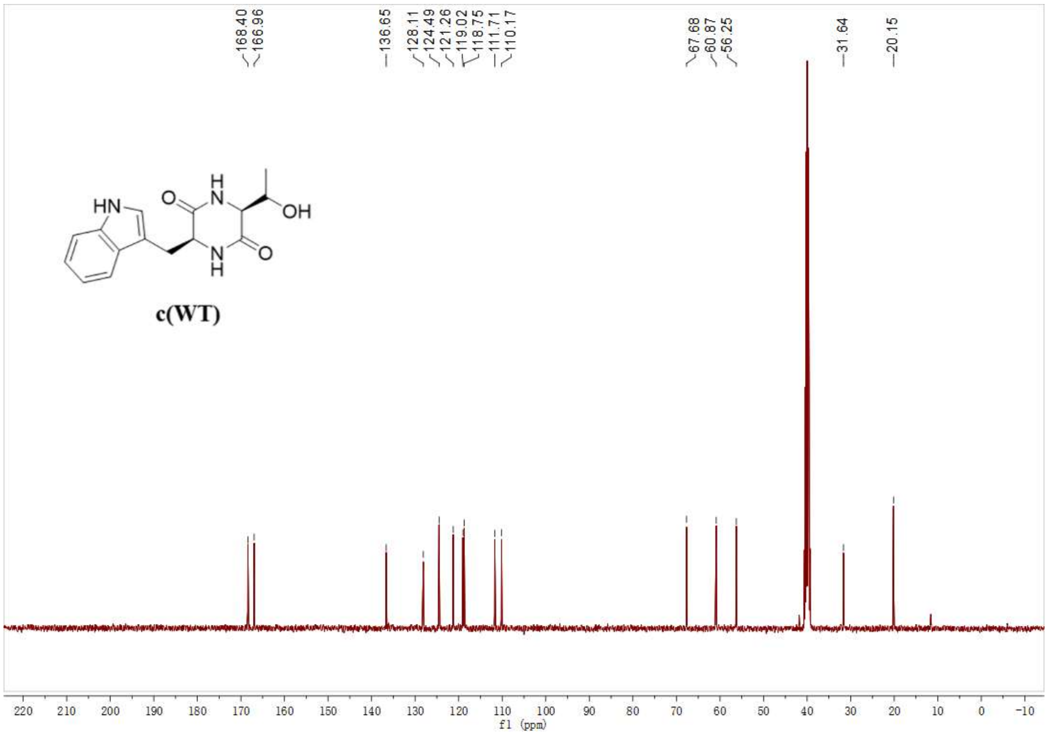

c(WE) - ^{1}H NMR Spectrum - DMSO-d_6, 400 MHz

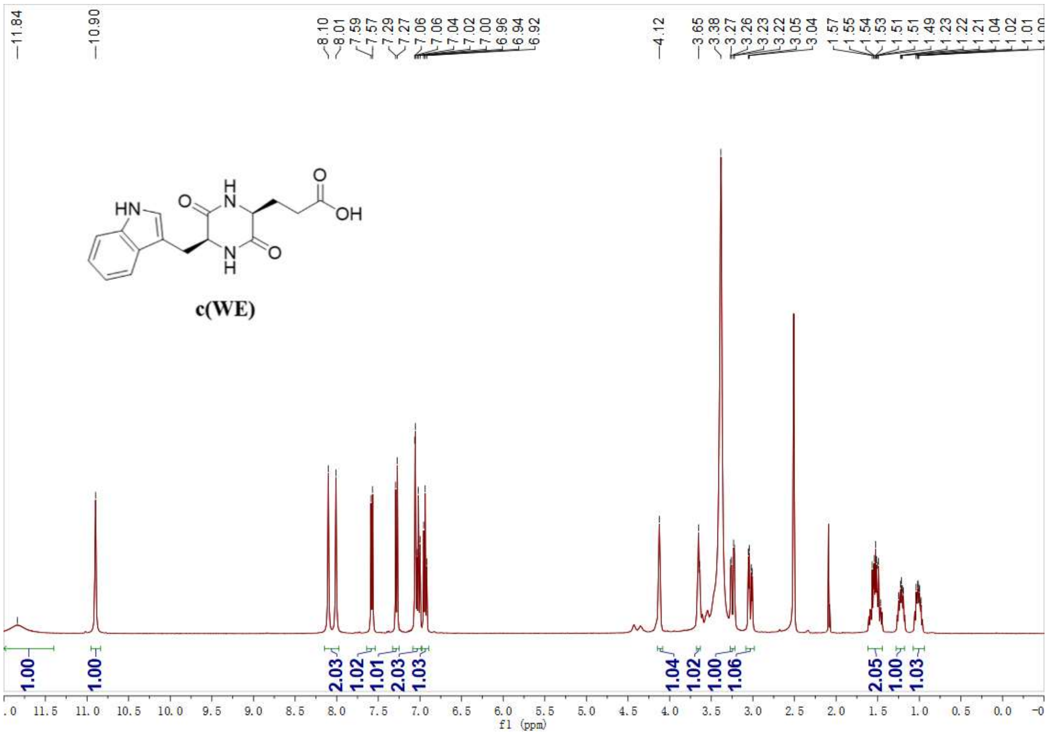

c(WE) – ^{13}C NMR Spectrum - DMSO-d_6, 100 MHz

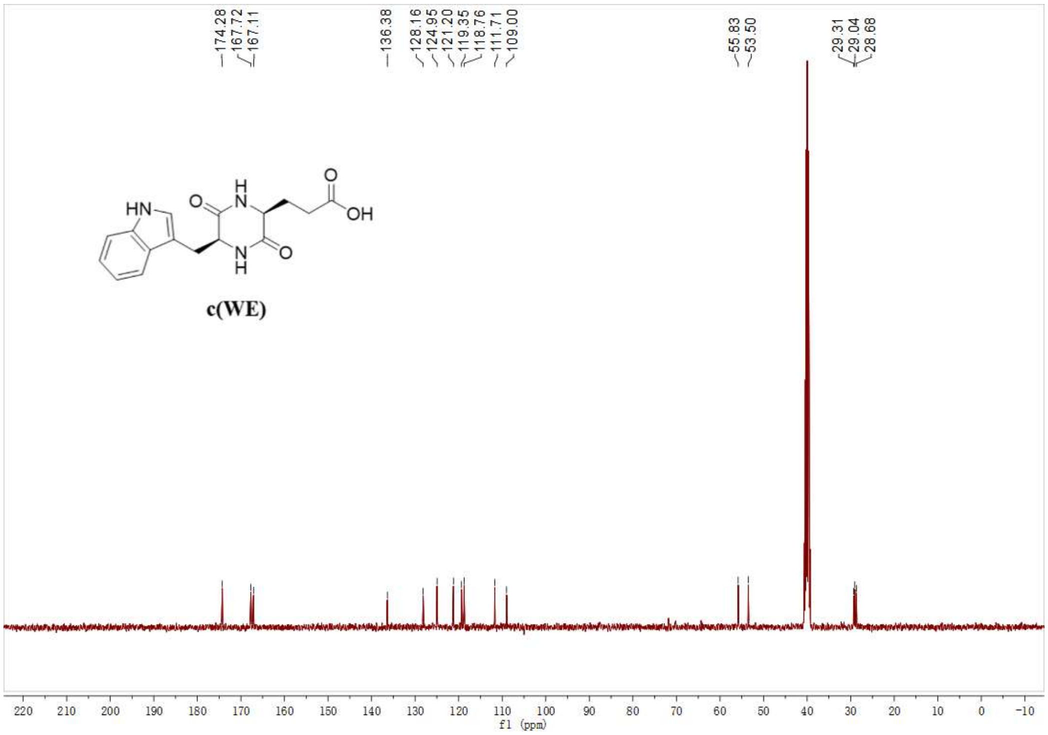

c(WK) - ^{1}H NMR Spectrum - DMSO-d_6, 400 MHz

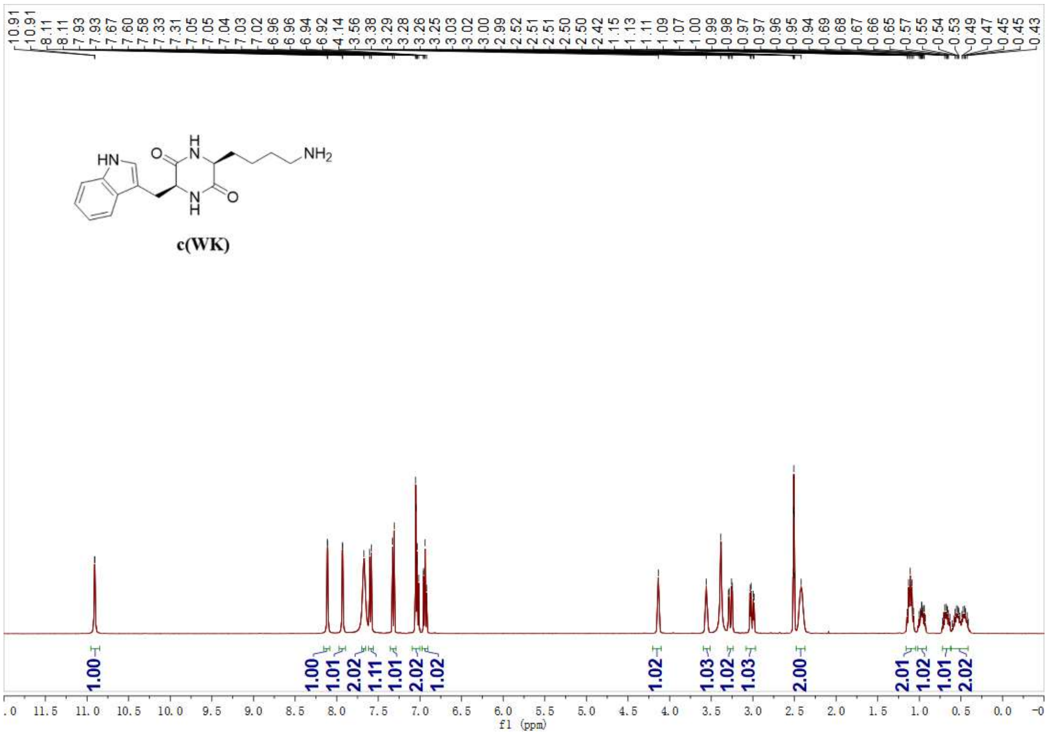

c(WK) – ^{13}C NMR Spectrum - DMSO-d_6, 100 MHz

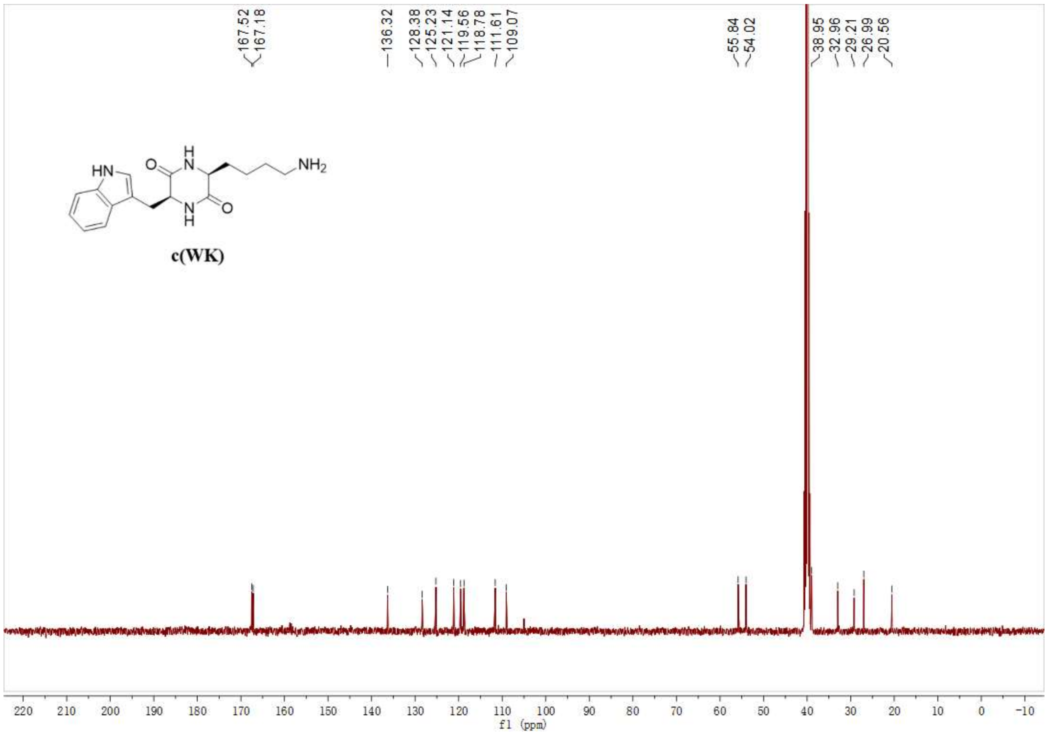

HPLC analysis of c(WS)

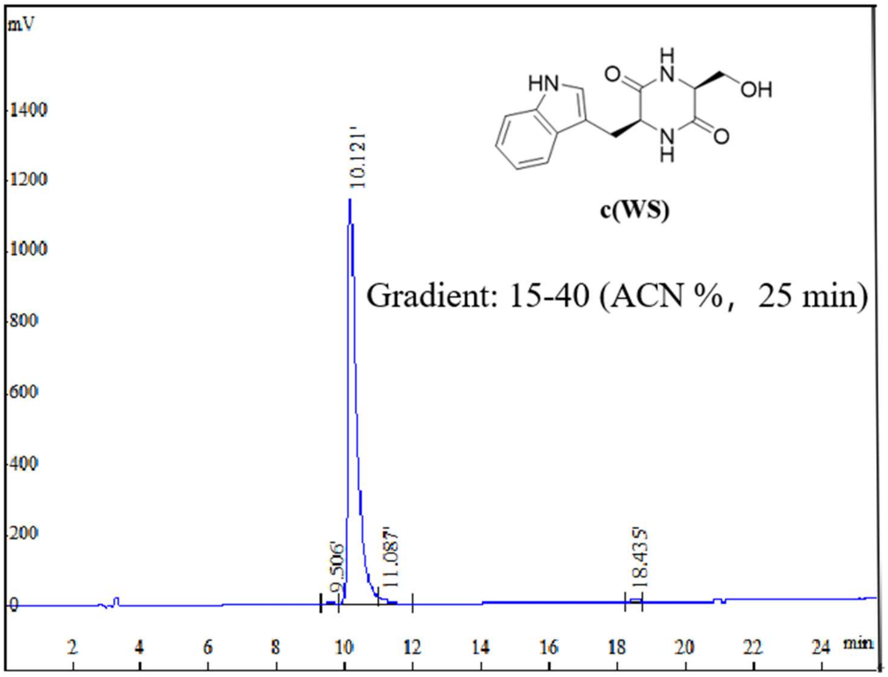

HPLC analysis of c(W*s*)

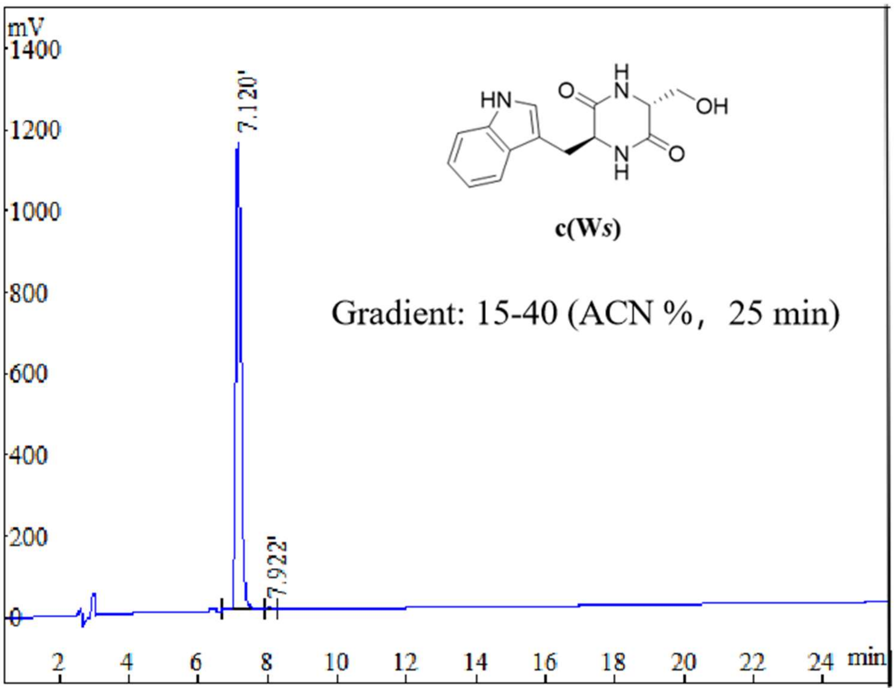

HPLC analysis of c(*w*S)

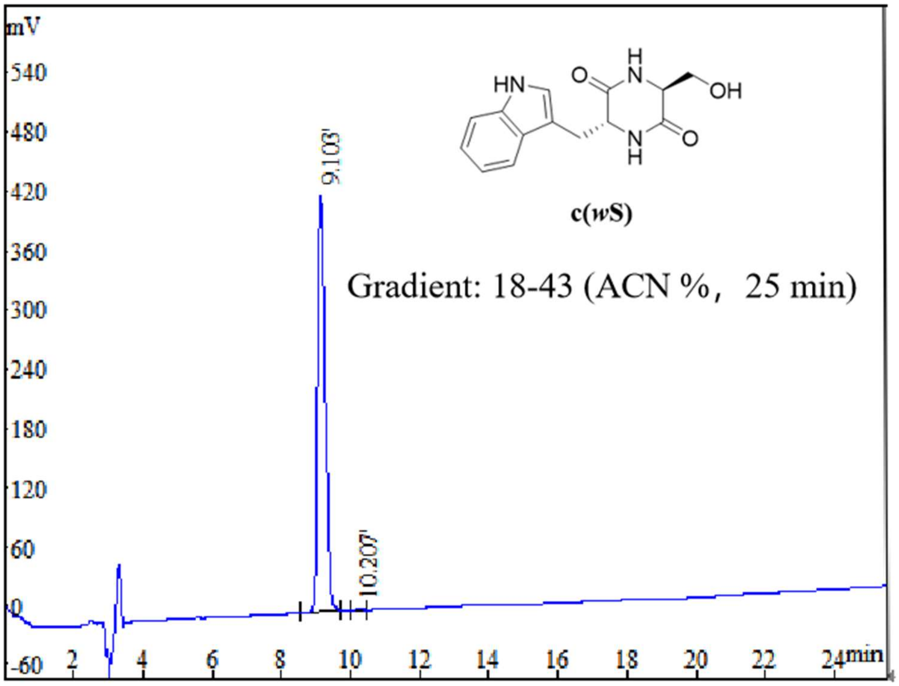

HPLC analysis of c(*ws*)

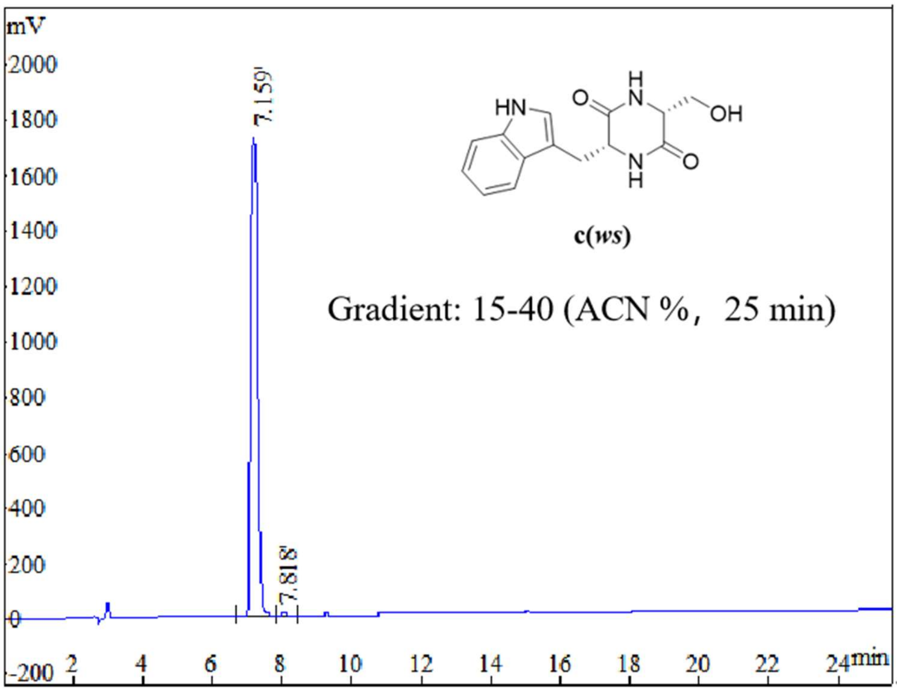

HPLC analysis of c(WA)

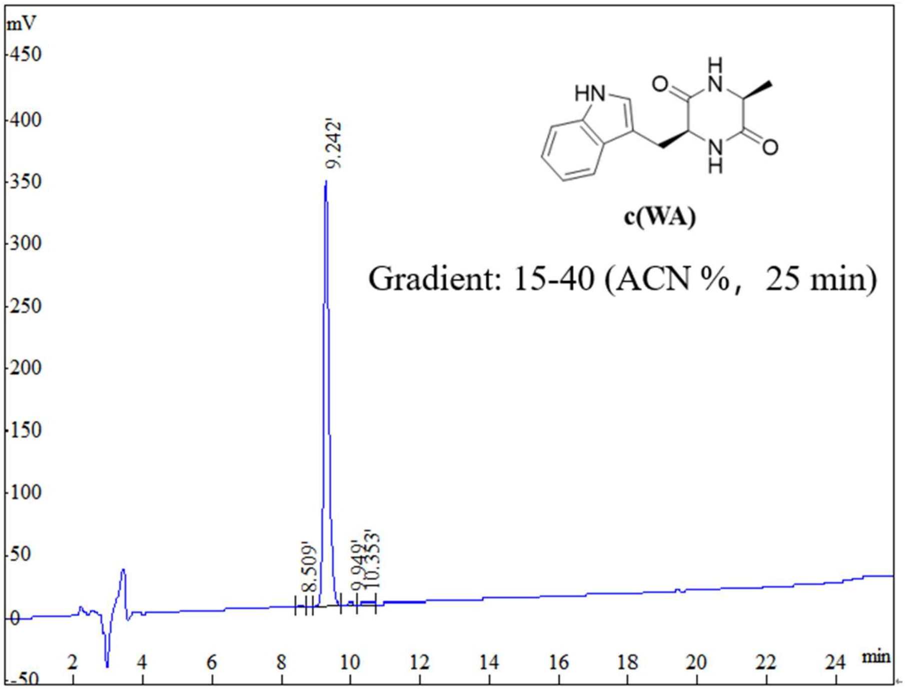

HPLC analysis of c(WT)

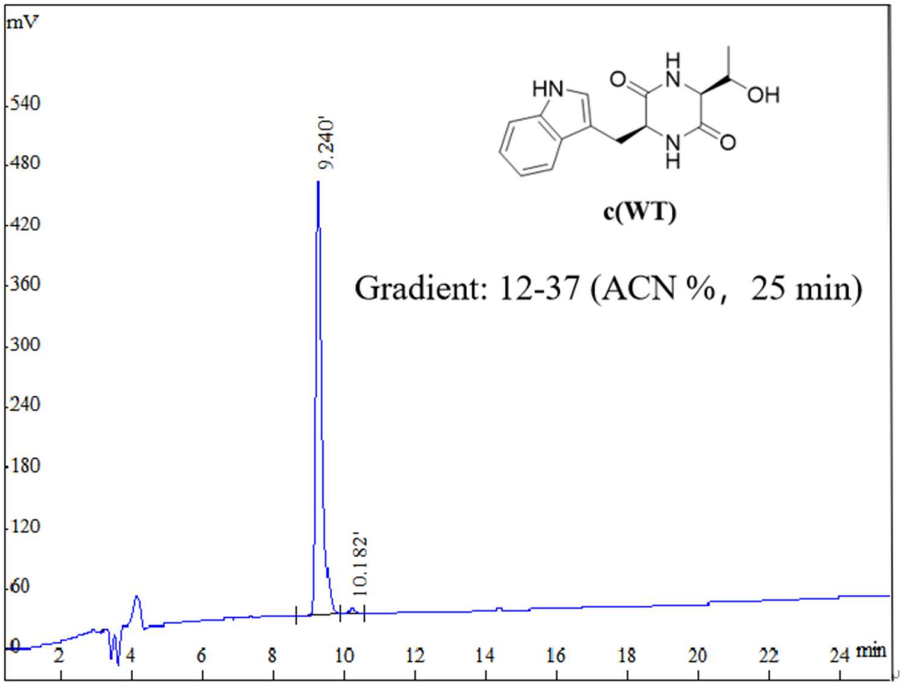

HPLC analysis of c(WE)

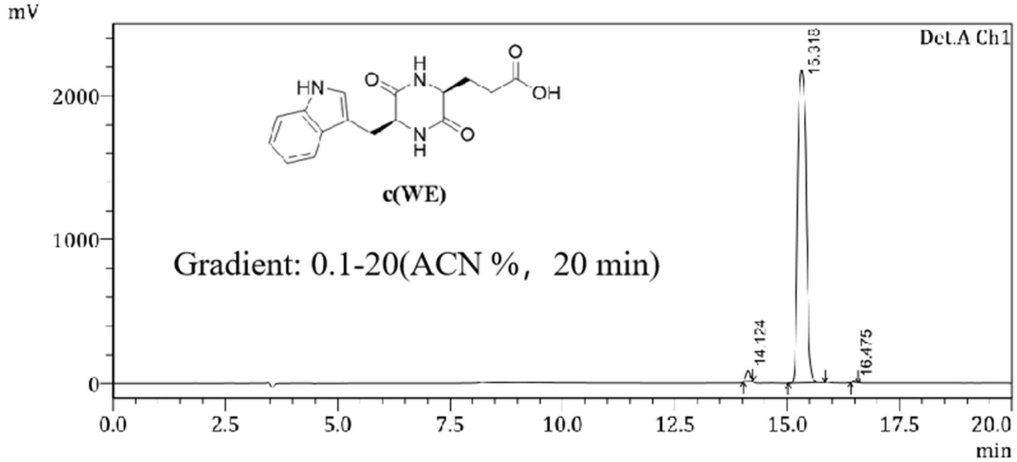

HPLC analysis of c(WK)

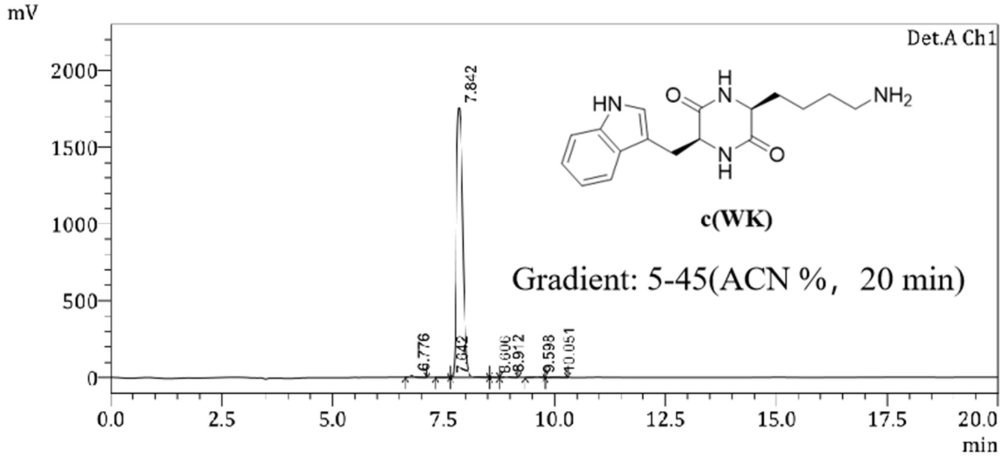

Supplement: Supplementary file 1 [file marinedrugs-20-00085-s001.zip › marinedrugs-1533756-supplementary.pdf]
